# Existing evidence on the effects of photovoltaic panels on biodiversity: a systematic map with critical appraisal of study validity

**DOI:** 10.1186/s13750-023-00318-x

**Published:** 2023-11-18

**Authors:** Alix Lafitte, Romain Sordello, Dakis-Yaoba Ouédraogo, Chloé Thierry, Geoffroy Marx, Jérémy Froidevaux, Bertrand Schatz, Christian Kerbiriou, Philippe Gourdain, Yorick Reyjol

**Affiliations:** 1 PatriNat (OFB (Office Français de la Biodiversité) – MNHN (Muséum National d’Histoire Naturelle)), 75005 Paris, France; 2 LPO (Ligue Pour la Protection Des Oiseaux), 17300 Rochefort, France; 3 CESCO (Centre d’Ecologie et des Sciences de la Conservation), UMR 7204, Muséum National d’Histoire Naturelle (MNHN), 75005 Paris, France; 4 CNRS (Centre National de la Recherche Scientifique), Sorbonne Université, Station Marine, 29900 Concarneau, France; 5 Biological and Environmental Sciences, University of Stirling, Stirling, FK9 4LA Scotland, UK; 6 CEFE, Université de Montpellier, CNRS, EPHE, IRD, 34090 Montpellier, France

**Keywords:** Conservation, Energy transition, Evidence synthesis, Floating solar, Green infrastructure, Ground-mounted photovoltaic, Solar panels, Wildlife

## Abstract

**Background:**

To phase out fossil fuels and reach a carbon–neutral future, solar energy and notably photovoltaic (PV) installations are being rapidly scaled up. Unlike other types of renewable energies such as wind and hydroelectricity, evidence on the effects of PV installations on biodiversity has been building up only fairly recently and suggests that they may directly impact ecosystems and species through, for instance, habitat change and loss, mortality, behaviour alteration or population displacements. Hence, we conducted a systematic map of existing evidence aiming at answering the following question: what evidence exists regarding the effects of PV installations on wild terrestrial and semi-aquatic species?

**Methods:**

We searched for relevant citations on four online publication databases, on Google Scholar, on four specialised websites and through a call for grey literature. Citations were then screened for eligibility in order to only retain citations referring to wild terrestrial and semi-aquatic species as well as PV and solar thermal installations, therefore excluding concentrated solar power. Accepted articles were first split into studies (corresponding to one experimental design) subjected to critical appraisal and then further split into observations (i.e. one population and one outcome) during metadata extraction. The current state of the literature was characterised and knowledge clusters and gaps identified.

**Review findings:**

Searching captured 8121 unique citations, which resulted in 158 relevant articles being accepted after screening. Even though the first article was published in 2005, the publication rate increased rapidly in 2020. The 97 included primary research and modelling articles were split into 137 unique studies and rated with either a low (43.8%), a high (41.6%) or an unclear overall risk of bias (14.6%) after internal validity assessment. Studies were further split into 434 observations, mainly carried out in the United States (23.0%) and the United Kingdom (21.0%), preferentially in temperate climates (64.5%). Plants and arthropods were the two most studied taxa (41.7% and 26.3%, respectively). Utility-scale solar energy (USSE) facilities were most often investigated (70.1%). Observations mainly focused on the effect of the presence of PV installations (51.8%). Species abundance, community composition and species diversity were the most common outcomes assessed (23.0%, 18.4% and 16.1%, respectively).

**Conclusions:**

Three knowledge clusters for which a systematic review should be contemplated were identified: (i) the effects of PV installations on plant and (ii) arthropod communities and, (iii) their effects at a larger ecosystem scale on overall species abundance. However, the currently available evidence regarding the effects of photovoltaic installations on biodiversity is still scarce. More research is urgently needed on non-flying mammals and bats as well as amphibians and reptiles. Solar thermal panels and floating PV installations should also be further investigated. Studies comparing different designs of PV installations, management practices or contexts should be conducted as well. Indeed, more evidence is still needed to allow decision-makers to accurately and reliably select the types of PV installations and management practices that are least damaging to biodiversity.

**Supplementary Information:**

The online version contains supplementary material available at 10.1186/s13750-023-00318-x.

## Background

Biodiversity erosion and climate change are the most pressing issues of the Anthropocene [1], and while both crisis are often treated separately, they have been shown to be interdependent [2]. Both crises should thus be tackled at the same time, as part of a more global strategy [1]. As global average temperatures and atmospheric concentrations of greenhouse gases are rising at an alarming rate, largely due to the combustion of fossil fuels for human energy needs [3], we need to rapidly phase out from carbon-intensive technologies and reach a carbon-neutral future. Alternative sources of energy are currently being rapidly scaled up, making the market of renewable energy technologies a thriving industry. In this context, solar photovoltaic power, one the most promising sources of renewable energy, accounted for over 60% of renewable electricity capacity additions worldwide in 2022, reaching a global capacity of more than a thousand gigawatt [4]. And its market share is expected to grow even further as the International Energy Agency (IEA) has forecast its installed power capacity to become the largest by 2027, even surpassing that of coal [5]. In the longer term, the IEA also estimates that the capacity of solar photovoltaic power would need to increase up to 20-fold to reach one fifth of global power supply, if the world were to reach a net zero CO_2_ future by 2050 [6].

Using solar energy to produce electricity is not a new concept and dates back to 1839 when Becquerel first discovered the photovoltaic effect [7]. Today, numerous technologies exist and allow us to benefit from the quasi-limitless pool of energy coming from the sun [8]. The most conspicuous ones are solar photovoltaic (PV) panels, which are comprised of semiconductors that convert sunlight into electricity. Several materials are used to build PV panels, from monocrystalline or polycrystalline silicon to heavy metals (e.g. copper indium selenide, cadmium telluride) [8]. Such PV panels can be installed on rooftops, in ground-mounted utility-scale facilities, which are often called Utility-Scale Solar Energy (USSE) facilities, or on water such as on the sea, lakes, reservoirs or canals [9–11]—often called floatovoltaics or floating PV/solar facilities. The sun’s energy can also be converted to heat by using solar thermal panels. These panels are usually used for household heating and installed on the top of roofs (or even as USSE facilities). They may either resemble regular photovoltaic panels or be made of tube solar collectors [8]. Concentrated Solar Power (CSP) plants, on the other hand, rely on an entirely different mechanism and usually consist of thousands of mirrors focusing sunrays into a central tower, which is thus heated at extreme temperatures. This heat is then used to produce vapour and electricity through a steam turbine, as in more common power generation facilities [8]. Nowadays, the expansion of CSP has been significantly hampered due to low policy support and its relatively higher costs compared to PV installations [5].

**Fig. 1:**
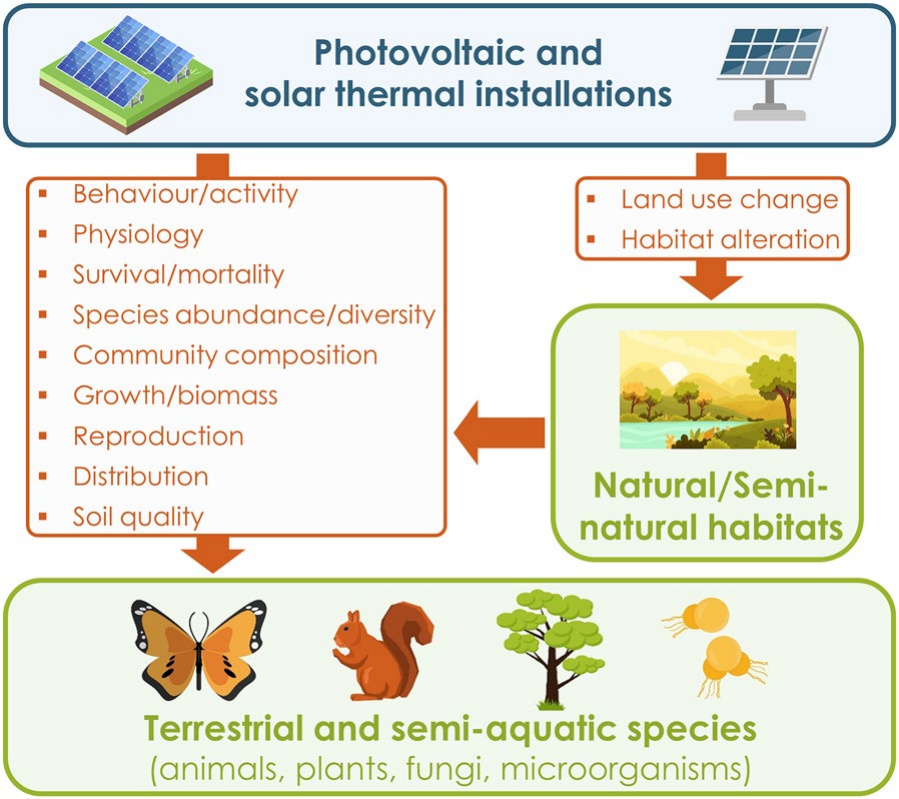
Conceptual model of the potential effects of photovoltaic and solar thermal installations on biodiversity. Orange arrows indicate the outcomes measuring the potential effects of photovoltaic and solar installations on terrestrial and semi-aquatic species and natural/semi-natural habitats. Created using images provided by Freepik

As solar energies are considered carbon neutral, or at least during their operational phase, they are anticipated to play a major role in replacing more traditional carbon-intensive power plants (such as coal- or oil-fired power plants) and could thus help cut global anthropogenic CO_2_ emissions; that is, if they do not actually lead to a rebound effect, which would eventually result in a greater energy consumption [12]. However, as with most types of human infrastructures [13–15], renewable energy installations may directly and indirectly harm wildlife and disturb natural ecosystems, either during the extraction of the necessary resources for their production, during their installation/dismantlement and/or during their operational phase, and can therefore contribute to declines in local biodiversity and, in turn, to the global biodiversity crisis. In particular, renewable energy installations may lead to significant changes and losses of natural habitats [16], often considered to be one of the most important driver of biodiversity erosion [1]. They may also represent a cause of bird and bat mortality, as it is increasingly recognised in the case of wind farms [17, 18], or impede fish migration routes and disrupt riparian ecosystems in the case of hydroelectric facilities [19]. As for solar energy, and more especially PV installations, while evidence has been building up only fairly recently due to its relatively new entry into the market of energy production, they have already been linked to a wide range of impacts on species and ecosystems such as land use change [20, 21], mortality [21, 22], disruption of plant growth [23, 24] and animal behaviour [25–27], changes of population composition and diversity [28–30] or alterations of soil quality and ecological functions [31] (Fig. 1). For instance, Horváth et al. [32] found that PV panels can reflect horizontally polarised light, which is often used by aquatic insects as a cue to detect water surfaces. The latter could then attempt to lay their eggs on these highly unsuitable surfaces, thus making PV panels potential ecological traps, which they hypothesised, might be responsible for local population declines near wetlands and water bodies. On a higher level of the food web, Kosciuch et al. [33] estimated that USSE facilities were responsible for 1.82 bird fatalities.MW^−1^.year^−1^ in the two states of California and Nevada, United States, which could represent a total of 30,976 bird fatalities per year when considering the total solar energy power capacity of both states (17.02 MW). At the community level, Graham et al. [34] found that plant bloom timing was delayed under partial shade from PV panels while floral abundance increased but pollinators were less abundant and diverse under full shade from PV panels. They linked these effects on plant and pollinator communities to alterations of microclimatic conditions under PV panels such as changes in soil temperature, solar radiation, or soil moisture—which can be directly related to nectar production by plants. Indeed, PV installations may even produce a photovoltaic heat island effect at the landscape scale with higher humidity levels and warmer night-time temperatures around USSE PV facilities [35, 36]. However, changes in microclimatic parameters could also turn into an opportunity for some plant species living in extremely arid ecosystems. Liu et al. [37] indeed observed increased plant biomass, coverage and richness within solar PV facilities compared to their reference, a sandy desert ecosystem. While the effects of PV installations have been primarily studied within ground-mounted USSE facilities, they may vary considerably according to the configuration of PV installations (i.e. whether on roofs, ground or water).

Apart from these reported effects of PV installations on biodiversity, other potential negative impacts have also been hypothesised in a certain number of reviews [10, 38, 39] and technical reports [40–44]. As such, PV installations might additionally generate chemical and noise pollution due to heavy machinery during their construction or operational phase [39], promote exotic species invasions through soil disturbances, lead to habitat fragmentation because of security fences surrounding USSE facilities or contribute to soil erosion and loss due to dust generation during construction and modified runoffs from PV panels [10, 38, 45]. However, many of these assumptions have been inferred and extrapolated from the impacts of other similar man-made infrastructures built in similar environments and thus, little empirical evidence is usually provided for the specific context of PV installations [46].

To our knowledge, despite the urgent and continuous need to disentangle the effects of PV installations on biodiversity, notably due to the current expansion of the PV market, no systematic map has been attempted so far. Moreover, for most reviews, authors rarely indicated their literature search strategies, nor did they provide an easily accessible database of the literature they collated, nor did they attempt to assess the internal validity of the studies they discussed. Hence, we propose this systematic map—which includes a critical appraisal of study validity—which synthesised the available evidence regarding the effects of PV and solar thermal (hereby both simply referred as PV) installations, whatever their scales (i.e. cells, panels, arrays, USSE facilities) and configurations (i.e. on roofs, ground or water), on wild terrestrial and semi-aquatic species. Indeed, we believe a comprehensive, transparent and objective evidence synthesis on this matter is needed in order to better inform decision-makers, guide future research and successfully contribute to biodiversity conservation while still mitigating anthropogenic climate change.

## Stakeholder engagement

This project was initiated following a call for proposal launched by the FRB (French Foundation for Research on Biodiversity) to conduct a systematic map concerning the impacts of human activities on terrestrial biodiversity. Our proposal to systematically map the available evidence regarding the effects of PV installations on biodiversity was accepted and thus, this project was granted funds from the FRB. The FRB is part of the steering committee which provided methodological expertise and followed the progress of this map all along the duration of the project. The FRB board is composed of 20 directors coming from eight French public research institutes as well as the corporate group LVMH, the Ineris (French National Institute for Industrial Environment and Risks), the University of Montpellier and the OFB (French Office for Biodiversity). The FRB’s main mission is to support and conduct research through scientific cooperation as well as to help increase and transfer knowledge on biodiversity-related issues.

A board of specialists on the matter of PV installations and their effects on biodiversity was identified and provided support to the review team when identifying the scope of the study, building the search string, defining the eligibility inclusion/exclusion criteria as well as assessing the validity of the metadata extraction codebook. This board was composed of experts coming from the French National Museum of Natural History (MNHN), the OFB, the French research Centre of Evolutionary and Functional Ecology (CEFE), the French National Centre for Scientific Research (CNRS), the University of Stirling as well as a French representative of BirdLife International (LPO).

## Objective of the map

This systematic map aims at synthesising the available evidence regarding the effects of PV installations, whatever their scales (i.e. cells, panels, arrays, USSE facilities), on biodiversity by building a comprehensive literature database and by highlighting any potential knowledge gaps or clusters, where, in the latter case, a systematic review could be contemplated.

### Primary question

The main question of this systematic map is: what evidence exists regarding the effects of PV installations, whatever their scales (i.e. cells, panels, arrays, USSE facilities), on wild terrestrial and semi-aquatic species?

### Component of the primary question

The above-mentioned primary question has the following Population–Exposure/Intervention–Comparator–Outcome elements (PECO, for the sake of clarity ‘exposure/intervention’ will be referred as ‘exposure’ beyond this point):

- Populations: All wild terrestrial and semi-aquatic species found globally (i.e. animals, plants, fungi, microorganisms living fully or partially in natural/semi-natural terrestrial habitats and ecosystems) were included. All natural/semi-natural habitats and ecosystems were considered while urban and agricultural habitats were discarded. Humans, domesticated and cultivated species as well as strictly aquatic ones (e.g. algae, fishes) were excluded.
- Exposures: All technologies of PV panels whatever their configurations (i.e. on roofs, ground, or water) were retained. All scales of PV installations were included whether it be cells, panels, arrays, or wider USSE facilities. Real and simulated experimental PV panels were both kept. The whole lifecycle of USSE facilities (i.e. construction, operation and dismantlement phases) was considered whereas the sole lifecycle of PV panels was excluded (i.e. material extraction, manufacturing and recycling phases). The interventions carried out at USSE facilities such as mowing, grazing or rehabilitation (with various types of seed mixes) were also included. Concentrated Solar Power (CSP) technologies were not included in this study. In addition, PV-powered devices such as global positioning tracking systems for animals, water pumps or lamps were considered to be out of the scope of this systematic map.
- Comparators: Studies comparing a population exposed to a PV installation and a population left unexposed and/or studies comparing a population before and after the construction of a PV installation were included (Control-Exposure spatial comparator and/or Before-After temporal comparator—e.g. BACE, BAE, CE). Studies comparing different types of PV installations (e.g. scale, inter-row width, height, angle, tracking system, technology) were considered as well. In addition, we also retained studies comparing different management techniques (e.g. mowing, grazing, rehabilitation) within USSE facilities as well as studies comparing different contexts (e.g. different climatic conditions or different ecosystems surrounding USSE facilities). Studies without any comparator were discarded.
- Outcomes: All outcomes related to the studied population were considered (e.g. mortality, diversity, abundance, growth, distribution, physiology, reproduction, mobility, morphology, behaviour, habitat alteration, habitat connectivity). All abiotic parameters related to the studied natural/semi-natural habitat or ecosystem were excluded.

### Secondary questions

In addition, this systematic map also aims at answering the following secondary questions:

1. What are the most studied species, habitats and ecosystems?
2. What are the characteristics of the studied PV installations (i.e. panel technology, size, fencing, type of management)?
3. Which types of outcomes are more usually investigated?
4. In which country and climatic zones have studies been carried out?
5. What level of reliability can be granted to the studies that are included in this systematic map?

## Methods

This systematic map followed the Collaboration for Environmental Evidence (CEE) guidelines for evidence synthesis in environmental management [47] and complied with ROSES RepOrting standards for Systematic Evidence Syntheses [48] (see Additional file 1). Even though the protocol for this map has been previously published in Environmental Evidence [49], a number of deviations from the methods initially proposed in the protocol emerged throughout the process of making this map and are acknowledged thoroughly in the following section.

### Deviations from the protocol

First, during citation retrieval, it appeared that the access to the OFATE (French and German Agency for Ecological Transition) database was restricted and therefore, no search could be performed on this specialised website.

Screening eligibility criteria were slightly refined in order to better match our mapping objectives. First, we more accurately defined our exclusion population criteria and thus, considered urban and agricultural habitats and ecosystems (i.e. areas in terms of land use/land cover) as being neither natural nor semi-natural. In addition, we considered the different interventions that can be carried out at USSE facilities such as mowing, grazing or rehabilitation (with various types of seed mixes) to be relevant to our research objectives. On the contrary, the exposure to PV-powered devices such as global positioning tracking systems for animals, water pumps or lamps was considered to be out of scope of our systematic map. Then, we decided to include two additional comparators at full-text screening: studies comparing different management techniques within USSE facilities and studies comparing different contexts—such as different climatic conditions or different ecosystems surrounding USSE facilities. Finally, document contents relative to Life Cycle Assessment (LCA) modelling studies were excluded.

As for metadata extraction, we expanded our codebook to better characterise the type of comparator, study design and control under scrutiny. As such, observations were further categorised following the type of comparator: whether it be relative to the presence and/or construction of PV installations, the type of PV installations being compared (e.g. scale, inter-row width, height, angle, tracking system, technology), the different management practices (e.g. mowing, grazing, rehabilitation) or different contexts being compared (e.g. location, surrounding ecosystems). Information on the availability and location of data within articles were extracted as well. Furthermore, we followed the more recent Köppen-Geiger climate classification provided by Rubel et al. [50] instead of the classification by Peel et al. [51] proposed in the protocol.

Finally, to improve metadata extraction and critical appraisal accuracy and quality, we tested reviewers’ agreement on a subset of 20% of accepted articles instead of the 5% proposed in the protocol. Study internal validity criteria were also slightly rephrased to further improve clarity and homogeneity with the CEE Critical Appraisal Tool (CEECAT) (see section ‘Study validity assessment’). As stated in the protocol [49], critical appraisal was performed on studies which corresponded to one experimental design. In this map, we refined the definition of a study, which thus corresponded to one experimental design; i.e. one exposure and one comparator.

### Search for literature

#### Languages

Searches were performed using exclusively English terms—which, based on the specificities of online publication database search engines, enable literature written in any other language to be retrieved. Only studies published in English or French were retained in this systematic map. We acknowledge that only including articles in these two languages constitutes a potential bias to our systematic map. Unfortunately, this could not be avoided based on the linguistic competences of the review team. The list of search terms is presented in the section below.

#### Search terms and string

A scoping exercise was carried out on the Web of Science Core Collection (WOSCC) database in order to build the search string. In order to reach the best search comprehensiveness and accuracy, several combinations of search terms describing populations and exposures were trialled iteratively (see protocol additional files [49]). Citations were finally retrieved using the following search string—which followed Web of Science format and was performed on topic ‘TS’:

TS = ((photovoltaic$ OR “solar panel$” OR “solar array$” OR “solar development$” OR “solar power” OR “solar park$” OR “solar installation$” OR “solar facilit*” OR “solar plant$” OR “utility-scale solar energ*” OR “utility scale solar energ*” OR biosolar OR “float* solar” OR floatovoltaic$) AND (biodiversity OR ecolog* OR ecosystem$ OR wildlife OR “natural habitat$” OR species OR flora OR vegetation$ OR animal$ OR fauna OR vertebrate$ OR mammal$ OR bird$ OR reptile$ OR amphibian$ OR invertebrate$ OR arthropod$ OR insect$ OR arachnid$ OR crustacean$ OR mollus* OR microbi* OR bacteri* OR microorganism$ OR fung*)).

#### Online publication databases

Using the access rights provided by the MNHN and the CNRS, we conducted the search on four multidisciplinary publication databases: Web of Science Core Collection (WOSCC), Biological Abstracts (BA), Zoological Records (ZR), all from Clarivate Analytics, as well as Scopus from Elsevier. All databases were selected for their relevance in the matter of ecological studies and for easy search reproducibility and accessibility. The WOSCC search included the following citation indices: Science Citation Index Expanded (SCI–EXPANDED, 1956–present), Social Sciences Citation Index (SSCI, 1975–present), Arts & Humanities Citation Index (A&HCI, 1975–present), Conference Proceedings Citation Index–Science (CPCI–S, 1990–present), Conference Proceedings Citation Index–Social Science & Humanities (CPCI–SSH, 1990–present), Book Citation Index–Science (BKCI–S, 2005–present), Book Citation Index–Social Sciences & Humanities (BKCI–SSH, 2005–present), Emerging Sources Citation Index (ESCI, 2017–present), Current Chemical Reactions (CCR–EXPANDED, 1985–present) and Index Chemicus (IC, 1993–present). As for BA, ZR and Scopus, we had access to all indexed databases (respectively 1969–present, 1864–present and 1788–present). Searches for WOSCC, BA and ZR were performed on 17 June 2022. We then adapted the abovementioned WOS search string to match the Scopus format for literature search (see Additional file 2). The search on the Scopus database was then carried out on 20 June 2022.

#### Internet searches

An additional search was carried out using Google Scholar on 28 June 2022 using the software Publish or Perish (v 8.2.3944, downloaded on 07 June 2022) [53]. The search string was simplified and divided into four to fit the search capabilities of this engine—limited Boolean operators and a maximum of 256 characters [54]. Searches were performed on titles exclusively. The first 250 hits of each search string were retained which resulted in 677 references being added to the literature database (see search strings and hits in Additional file 2).

#### Specialist sources

In addition, we searched for relevant citations on the following specialist websites (English or French):

- IEA (International Energy Agency): https://www.iea.org/
- IRENA (International Renewable Energy Agency): https://www.irena.org/
- United States Office of Energy Efficiency and Renewable Energy: https://www.energy.gov/eere/office-energy-efficiency-renewable-energy
- ADEME (French Agency for Ecological Transition): https://www.ademe.fr/

Searches were carried out on 20 and 21 October 2022 and added 20 additional references to our literature database (see Additional file 2). We could not search for any citations on the OFATE (French and German Agency for Ecological Transition) website due to restricted access rights.

#### Supplementary searches

A call for literature was conducted through a professional network to find non-peer reviewed literature in English and/or French—i.e. technical reports, MSc or PhD theses. The call was initiated on 6 September 2022 and provided 234 additional references to our literature database (see Additional file 2). Finally, 8 relevant citations not retrieved by the literature search were added after being identified by the review team throughout the mapping process (see Additional file 2).

#### Estimating the comprehensiveness of the search

A test list of 26 relevant primary research articles was established in order to assess the comprehensiveness of the literature search. These articles were identified by the review team, with the help of experts or through previous syntheses on PV installations and biodiversity carried out by French operational actors such as the ADEME (French Agency for Ecological Transition) [42], the LPO [43] as well as the IUCN (International Union for Conservation of Nature) [40].

Among the 26 articles of the test list, 25 were indexed in WOSCC and 25 in Scopus—both thus provided a 96% indexation level, indicative of a high degree of relevance of these two databases for our literature search. As for BA, it had 18 indexed articles belonging to the test list (72%). On these three databases, one article from Bousselot et al. [52] was consistently not indexed. We thus checked its presence on ZR but it was not indexed in this database either. Among the 26 articles of the test list, our search string retrieved 100% of the articles indexed in WOSCC (25 articles), in Scopus (25 articles) as well as in BA (18 articles). Details on search hits from each selected database can be found in Additional file 2.

#### Assembling and managing search results

The results of all previous searches were collated (see Additional file 2). Then, duplicate removal was carried out manually through duplicate conditional formatting and visual identification with Microsoft Excel software. Full-texts were retrieved automatically with the reference management software Endnote or otherwise manually. In addition, the services of the MNHN library helped us find several other full-texts which had proved more difficult to retrieve.

### Article screening and study eligibility criteria

#### Screening process

After duplicate removal, citations were screened for eligibility on title and subsequently on full-text. For both title and full-text screening stages, reviewers’ decision consistency was assessed following a two-step process: first, a small sample of references was randomly selected and independently screened by all three reviewers; then, all reviewers met and resolved their disagreements, and eligibility criteria clarified and refined if judged necessary. A Randolph’s Kappa (κ) coefficient [55] was also computed. If the Randolph’s κ was superior to 0.7, we considered the level of agreement between reviewers to be acceptable and therefore proceeded to a second similar test on a larger subset of references. Otherwise, if the Randolph’s κ was inferior to 0.7, the process was repeated from the start. In the end, the consistency of decisions between reviewers was assessed on a total subset of at least 10% of references from our literature corpus—actually, 10.5% for title screening and 10.2% for full-text screening. This proportion results from a compromise between high volumes of citations and time constraints and has usually been chosen in recent systematic maps and reviews—albeit the best and optimal practice would be, for all citations, to be screened by at least two reviewers [56]. After sufficient agreement was ensured, each reviewer then independently performed title or full-text screening on different subsets of the corpus. Special care was taken to ensure no reviewer ever had to screen articles they co-authored.

As a whole, title screening was performed by the three reviewers CT, RS and AL whose decision consistency was checked on a subsample of 850 citations out of the 8121 of the de-duplicated corpus (10.5%). The first Randolph’s κ test yielded a coefficient of 0.70 (200 references) and the second 0.92 (650 references). Full-text screening was carried by the three reviewers CT, DYO and AL whose decision consistency was checked on a subsample of 99 citations out of the 974 retrieved full-texts (10.2%). The first Randolph’s κ test yielded a coefficient of 0.91 (30 full-texts) and the second 0.79 (69 full-texts).

#### Eligibility criteria

At the title screening stage, the eligibility of citations was assessed on population–exposure–outcome criteria (Table 1). At the full-text screening stage, complete population–exposure–comparator–outcome criteria were used as well as additional language, document type and content criteria.

**Table 1:** List of eligibility criteria used at title and full-text screening

Include	Exclude
Populations	Populations
- All wild terrestrial and semi-aquatic species found globally (i.e. animals, plants, fungi, microorganisms living fully or partially in natural/semi-natural terrestrial habitats and ecosystems) - All natural/semi-natural habitats and ecosystems (i.e. areas in terms of land use/land cover)	- Humans - Domesticated or cultivated species - Strictly aquatic or marine species (microalgae, fishes) - Urban and agricultural habitats and ecosystems (i.e. areas in terms of land use/land cover)
Exposures	Exposures
- All technologies of PV panels (e.g. monocrystalline, CdTe) whatever their configurations (i.e. on roofs, ground, or water) - Real or simulated PV panels - All scales of PV installations whether it be cells, panels, arrays, or wider USSE facilities - The whole lifecycle of USSE facilities (i.e. construction, operation and dismantlement phases) - Management practices being carried out at USSE facilities (e.g. mowing, grazing, rehabilitation)	- PV-powered devices such as global positioning tracking systems for animals, water pumps or lamps - CSP - The lifecycle of PV panels (i.e. material extraction, production and recycling phases)
Outcomes	Outcomes
- All outcomes related to the studied population (e.g. mortality, diversity, abundance, growth, distribution, physiology, reproduction, mobility, morphology, behaviour, habitat alteration, habitat connectivity, etc.)	- All abiotic parameters related to the studied natural/semi-natural habitat or ecosystem (e.g. humidity, temperature, radiation)
Comparators	Comparators
- Studies comparing a population exposed to a PV installation and a population left unexposed and/or studies comparing a population before and after the construction of a PV installation—Before-After temporal comparator and/or Control-Exposure spatial comparator (e.g. BACE, BAE, CE) - Studies comparing different types of PV installations (e.g. technology, size, inter-row, orientation, angle) - Studies comparing different management practices within USSE facilities (e.g. mowing, grazing, rehabilitation) - Studies comparing different contexts such as different climatic conditions or different ecosystems surrounding USSE facilities	- Studies without any comparator
Languages	
- Articles written in English or French	
Document types	Document types
- Journal article, book chapter, technical report, PhD or MSc theses	- Conference objects (e.g. meeting abstracts, slides, posters)
Document contents	Document contents
- Primary research articles, reviews, meta-analyses, modelling studies without experimental data	- LCA modelling studies

First, for title screening, citations were only checked for population–exposure–outcome criteria. Then, complete population–exposure–comparator–outcome criteria as well as language, document type and content criteria were used for full-text screening

*PV* Photovoltaic and solar thermal; *USSE* Utility-Scale Solar Energy; *CSP* Concentrated Solar Power; *LCA* Life Cycle Assessment

Strictly aquatic species were excluded based on the demands of the stakeholders who commissioned this systematic map. However, as floating PV installations may also impact aerial, terrestrial or semi-aquatic species such as birds, insects or amphibians, floatovoltaics were considered as a valid exposure. We acknowledge that CSP may also be a substantial threat for biodiversity and that the evidence regarding their impacts should be summarised as well [10]. Nevertheless, as this technology relies on mirrors to collect solar energy and not on panels, we considered that both exposures were too different and therefore excluded CSP. Regarding natural/semi-natural habitats and ecosystems, we only focused on biotic outcomes resulting from PV installations (e.g. lost area for wildlife) but citations strictly dealing with modifications of abiotic parameters (e.g. humidity, temperature, radiation) were excluded. In addition to what was initially stated in the protocol [49], the different management practices being carried out at USSE facilities such as mowing, grazing or rehabilitation (with various types of seed mixes) were considered to be a relevant intervention for our research objectives while exposures to PV-powered devices such as global positioning tracking systems for animals, water pumps or lamps were judged to be out of scope and therefore excluded. Subsequently, studies comparing different management practices within USSE facilities (e.g. mowing, grazing, rehabilitation) as well as studies comparing different contexts such as different climatic conditions or different ecosystems surrounding USSE facilities were included.

We considered all possible contents being primary research, reviews, meta-analyses or modelling studies but discarded LCA modelling studies. Reviews and meta-analyses were separated from the main literature database and their metadata coded in another datasheet. Conference objects (e.g. meeting abstracts, slides, posters) were excluded because of their relatively low content in useful data and information. The list of excluded citations at the full-text stage alongside reasons for exclusion is provided in Additional file 2.

### Study validity assessment

All primary research articles accepted after screening were split into studies—one study referring to one experimental design: i.e. one exposure and one comparator—and each study was submitted to internal validity assessment.

A Critical Appraisal Tool (CAT) was developed by the review team based on the criteria identified in the CEECAT [57]—i.e. Confounding factors, Post-exposure selection, Misclassified comparison, Performance, Detection, Outcome reporting and Outcome assessment risks of bias. This CAT allowed the assessment of both experimental and observational primary research studies. We also added a supplementary exposure risk of bias criterion which assessed whether experiments were carried out on simulated PV panels—for example, plastic sheeting on wood panels [58]. Even though such studies were within the scope of our systematic map, we wanted to take into account the potential high levels of confounding factors and thus high risk of bias, notably regarding the differences of microclimatic conditions between simulated and real PV panels. We chose to adapt CEECAT questions and decision trees to better match the context of this map and because of time constraints. As our internal validity assessing questions could be answered by a binary Yes or No, we only assigned studies with a low or high risk of bias rating—as well as an unclear rating for studies with insufficiently accurate or unknown information [56] (see Table 2, Additional file 3). In the end, a study’s overall risk of bias was classified as low if all criteria were rated with a low risk of bias, unclear if at least one criterion was rated with an unclear risk of bias and high if at least one criterion was rated with a high risk of bias. Reviews and meta-analyses were not submitted to any critical appraisal. Studies’ external validity was not evaluated in this systematic map as we only assessed the validity of the general knowledge base on the effects of PV installations on biodiversity and did not attempt to answer a precise systematic review question.

**Table 2:** Studies internal validity critical appraisal tool

Risk of bias	Question	Low	High	Unclear
Confounding factors	Are there potential confounding factors influencing the exposure and/or outcome? (e.g. different ecosystems between sites, additional uncontrolled exposures such as light, chemical or noise pollution)	No or Seemingly no	Yes or Seemingly yes	Unknown or Unclear
Post-exposure selection	Are exposure and comparator groups randomly or systematically selected and exchangeability can be assumed after the exposure?	Yes or Seemingly yes	No or Seemingly no	Unknown or Unclear
Attrition	Were there any differences in missing data between exposure and comparator groups during the study or the analysis?	No or Seemingly no	Yes or Seemingly yes	Unknown or Unclear
Misclassified comparison (only for observational studies)	Are exposure and comparator groups sufficiently well defined?	Yes or Seemingly yes	No or Seemingly no	Unknown or Unclear
Performance (only for experimental studies)	Was the exposure altered during the experiment and thus differed between exposure and comparator groups?	No or Seemingly no	Yes or Seemingly yes	Unknown or Unclear
Detection	Are they differences in how outcomes were measured between exposure and comparator groups?	No or Seemingly no	Yes or Seemingly yes	Unknown or Unclear
Outcome reporting	Are reported findings selectively disclosed?	No or Seemingly no	Yes or Seemingly yes	Unknown or Unclear
Outcome assessment	Were assumptions for the applied statistical analyses violated? (e.g. normality, homoscedasticity)	No or Seemingly no	Yes or Seemingly yes	Unknown or Unclear
Exposure	Are real (not simulated) photovoltaic or solar thermal panels used?	Yes or Seemingly yes	No or Seemingly no	Unknown or Unclear

Adapted from the Collaboration for Environmental Evidence Critical Appraisal Tool (CEECAT) [57]

Before beginning critical appraisal, a random subset of 20 accepted articles (20.6%) was assessed by the two reviewers DYO and AL in three successive steps—corresponding to 35 studies when split by experimental designs; i.e. one exposure and one comparator. Reviewers then met to discuss and resolve all possible disagreements. Finally, all remaining studies were independently critically appraised by AL. At the end of the internal validity assessment stage, DYO cross-checked 6 articles critically appraised by AL (6.2%)—corresponding to 11 studies. Special care was taken to ensure no reviewer ever had to critically appraise articles they co-authored. The full process of internal validity assessment with results and justifications is provided in Additional file 3 and overall risk of bias are also appended to each study and subsequent observations in Additional file 4.

### Data coding strategy

As one study (corresponding to one experimental design; i.e. one exposure and one comparator) can investigate several different populations and/or outcomes, all primary research studies were further split into observations—each observation referring to one population and one outcome. All metadata for each observation were coded in a coding form according to a pre-identified list of relevant variables (see codebook in Additional file 4). The key variables included:

- Bibliographic information (article, study and observation unique identifiers, authors, title, year, journal, DOI, language, document type, document content)
- Review information (reviewer, study internal validity assessment result)
- Description of study design (e.g. location, country ISO code, climatic zone, number of sample sites)
- Description of comparator (e.g. type of comparator, type of study design, description of the control)
- Description of population (e.g. species, taxonomic group)
- Description of exposure (e.g. technology, size, context, management)
- Description of the different types of outcomes related to survival/mortality, diversity, abundance, composition, behaviour, physiology, distribution, biomass, reproduction, activity, ecosystem fluxes, presence, land use, habitat alteration, habitat connectivity
- Availability and location of data within articles

**Fig. 2:**
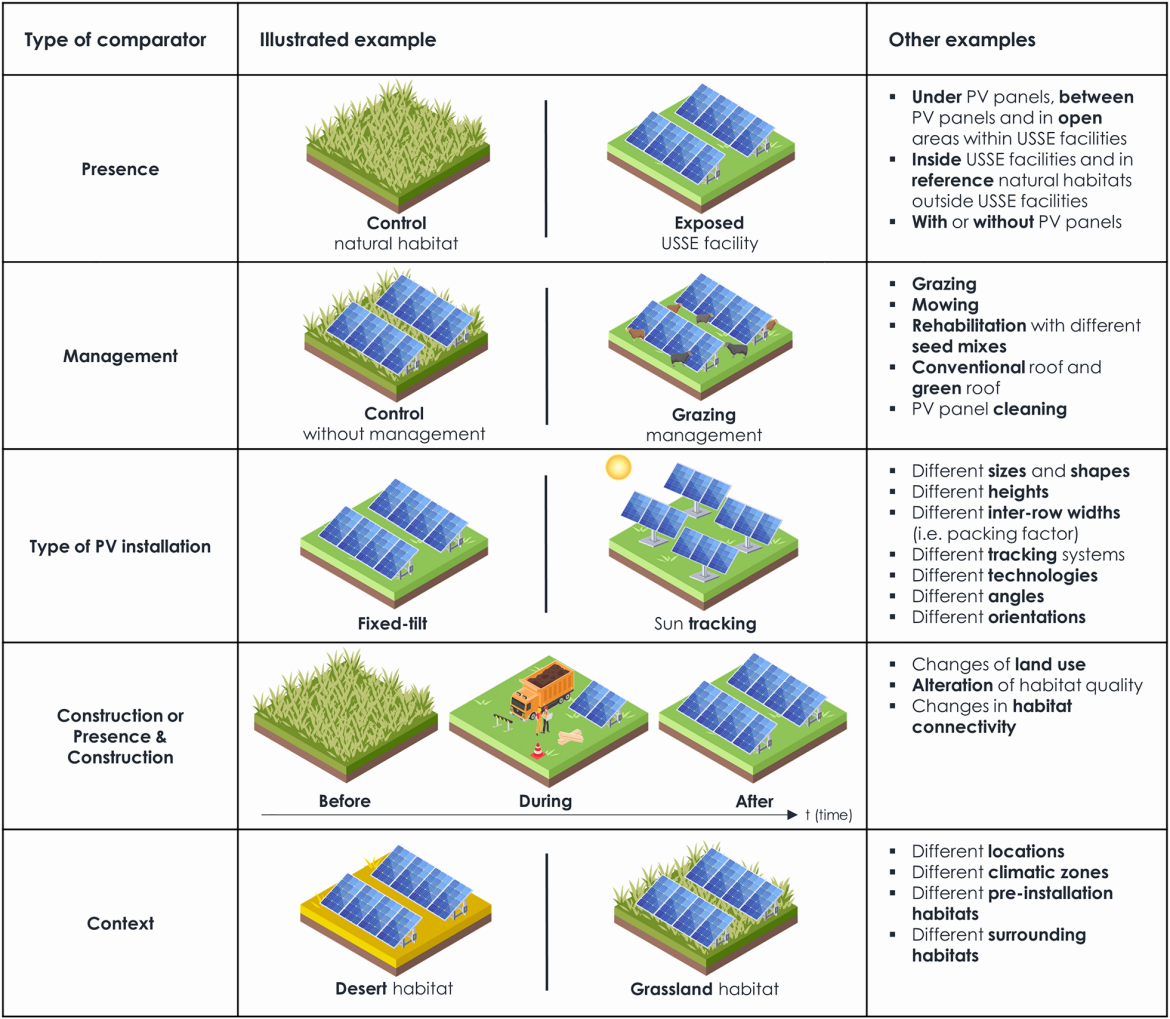
The different types of comparators used during metadata extraction. *PV* Photovoltaic and solar thermal; *USSE* Utility-Scale Solar Energy. Created using images provided by Freepik

Climatic zones were identified according to the Köppen-Geiger climate classification which was displayed on a Google Earth layer [50]. Metadata coding was conducted by the two reviewers YR and AL. Before beginning the metadata extraction stage, reviewers’ level of agreement was discussed and resolved on a random subset of 20 articles (20.6%)—following four subsequent steps, each consisting of 5 articles and corresponding to a total of 92 observations when split by population and outcome. These discussions allowed the metadata codebook to be further clarified and expanded through the addition of new variables which were judged as missing from the initial codebook proposed in the protocol [49]—e.g. further description of study design and type of comparator (Fig. 2), availability and location of data. Then, AL independently extracted the metadata for all remaining articles. At the end of the metadata extraction phase, YR cross-checked 5 articles extracted by AL (5.2%)—corresponding to 35 observations. Special care was taken to ensure no reviewer ever had to extract metadata from articles they co-authored.

### Data mapping method

An open-access database of all observation data with associated study-level overall risk of bias was produced alongside this systematic map report (see Additional file 4). A geographic map, tables and bar charts were used to represent the distribution of key variables extracted during metadata extraction—i.e. source, document type and content, year of publication, location, internal validity assessment result, population, exposure, comparator, outcome. In addition, heat maps showing the distribution of observations by population, comparator and outcome were produced in order to identify key knowledge clusters and gaps. In the case of this map, an arbitrary threshold of 100 observations was chosen to identify the main knowledge clusters, deemed to be sufficiently well represented for a future systematic review to be considered. The identified knowledge clusters and gaps were subsequently used to formulate recommendations for policy-makers and researchers.

All statistical analyses were carried out on the R software (version 4.3.1) [59] and graphs were customized with the ‘ggplot2’ package [60].

## Review findings

### Review descriptive statistics

#### Results of the search for literature

**Fig. 3:**
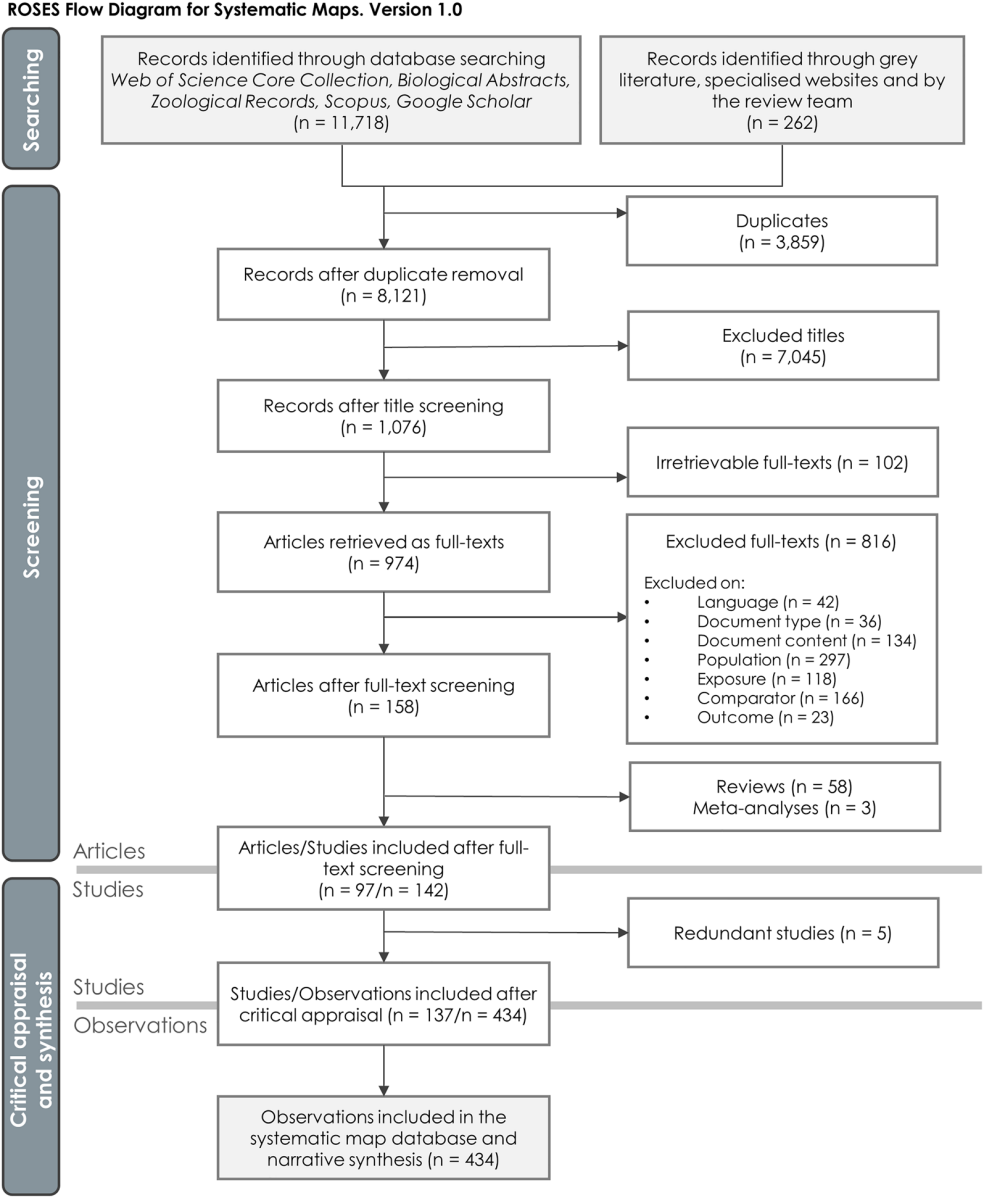
ROSES flow diagram reporting the screening process of the articles, studies and observations included in the systematic map [67]

Searches on online publication databases yielded 3,978 citations for Web of Science Core Collection, 1,012 for Biological Abstracts, 102 for Zoological Records and 6,130 for Scopus. Additional searches on Google scholar and specialised websites provided respectively 677 and 20 citations. Finally, the call for grey literature provided an addition of 234 references and the review team also provided 8 relevant citations which were not otherwise retrieved (see Additional file 2). Out of the overall 11,980 retrieved citations, 8,121 unique references were kept after duplicate removal (Fig. 3). Title screening resulted in 1,076 citations being accepted, for which we were able to collect 974 full-texts. The remaining 102 missing full-texts (9.5%) were either unavailable to us or could simply not be found. After full-text screening, 158 relevant articles were retained which corresponded to 92 primary research articles, 5 modelling articles, 58 reviews and 3 meta-analyses. Full-texts were mainly excluded due to irrelevant populations (36.4%), comparators (20.3%), contents of document (16.4%) or exposures (14.5%). It should be noted that, during full-text screening, a group of articles using PV-powered devices such as animal global positioning tracking systems, water pumps or lamps [61–66] was excluded on the exposure criterion because we considered the population was rather exposed to treated water or light rather than strictly to a PV installation. The whole screening process as well as excluded full-texts with reasons for exclusion are provided in Additional file 2.

#### Sources and document types of included articles

**Fig. 4:**
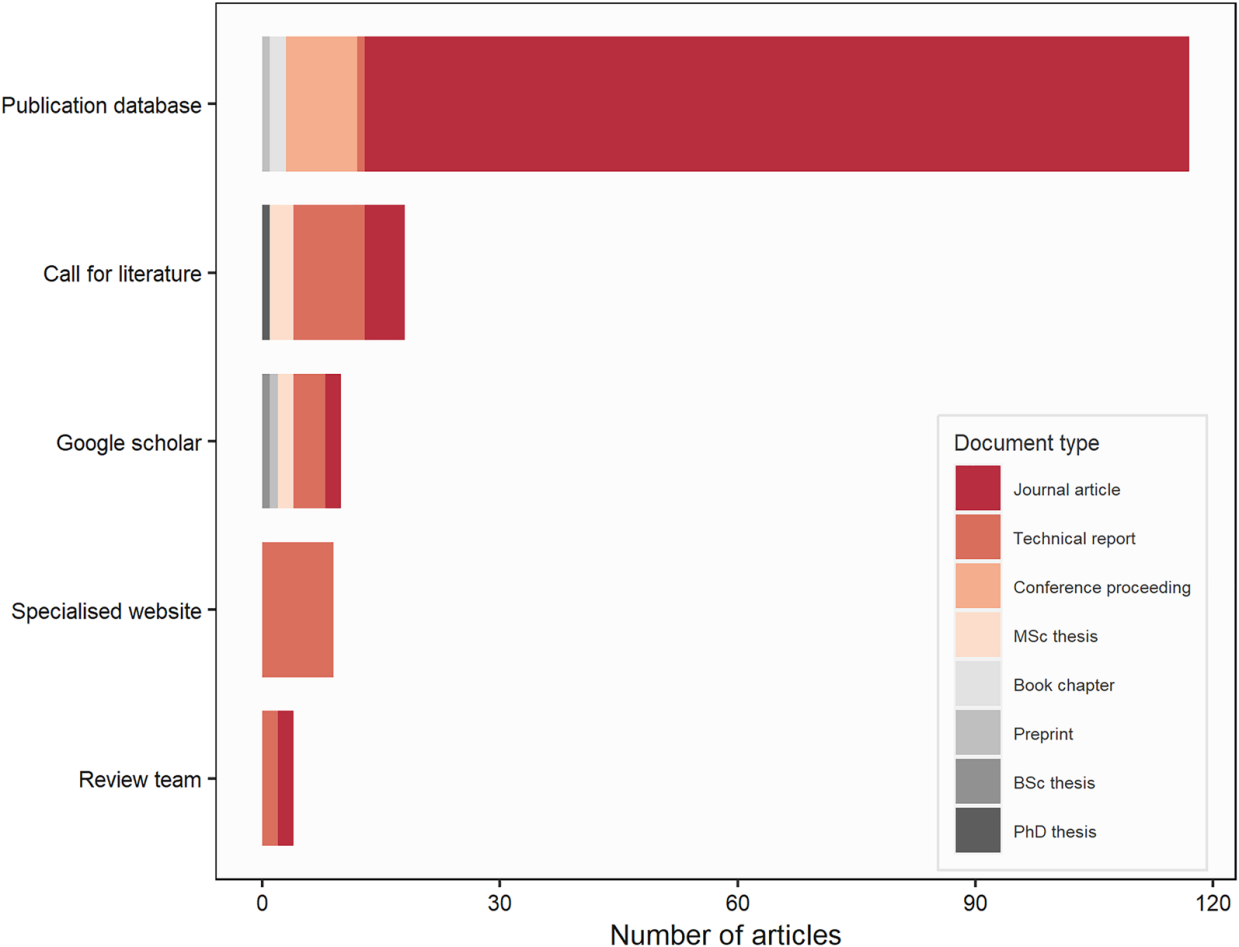
Number of included articles by source and type of document

About three quarters of accepted articles were retrieved by the main searches on online publication databases (117 articles, 74.1%) (Fig. 4). The searches on Google scholar yielded 10 additional articles (6.3%). Then, the call for grey literature provided a substantial number of supplementary references with 18 articles (11.4%). Searches on specialised websites resulted in 9 articles (5.7%) being accepted. Finally, four additional articles (2.5%), which were found by the review team but had not been otherwise retrieved, were also included.

Accepted articles consisted mainly of journal articles (71.5%) and were more often written in English (92.4%). In addition, 25 technical reports (15.8%), 9 conference proceedings (5.7%), five MSc theses (3.2%) as well as one PhD and one BSc theses (each 0.6%), two preprints (1.3%) and two book chapters (1.3%) were found.

#### Chronological distribution and document contents of included articles

**Fig. 5:**
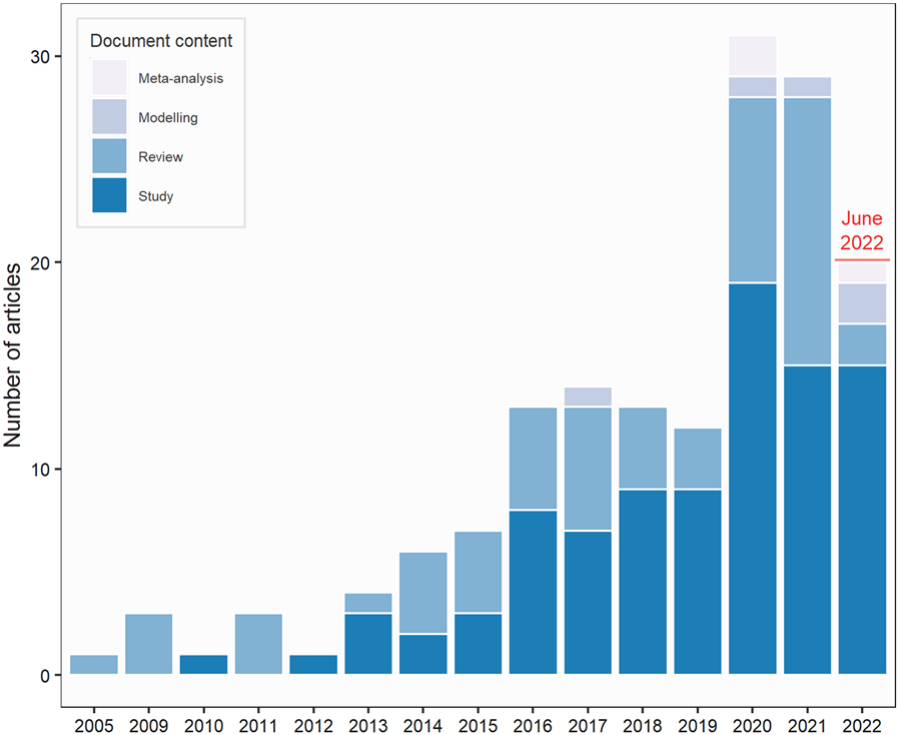
Number of included articles by year of publication and content of document. As the search was conducted in June 2022, it may not be fully representative of the final volumes published that year

The first published article dated back to 2005 (Fig. 5) but the publication of primary research articles and reviews have truly accelerated since 2015—it has to be noted that, as the search was performed in June 2022, it may not be fully representative of the final volumes published that year.

#### Distribution of included studies by internal validity assessment results

**Fig. 6:**
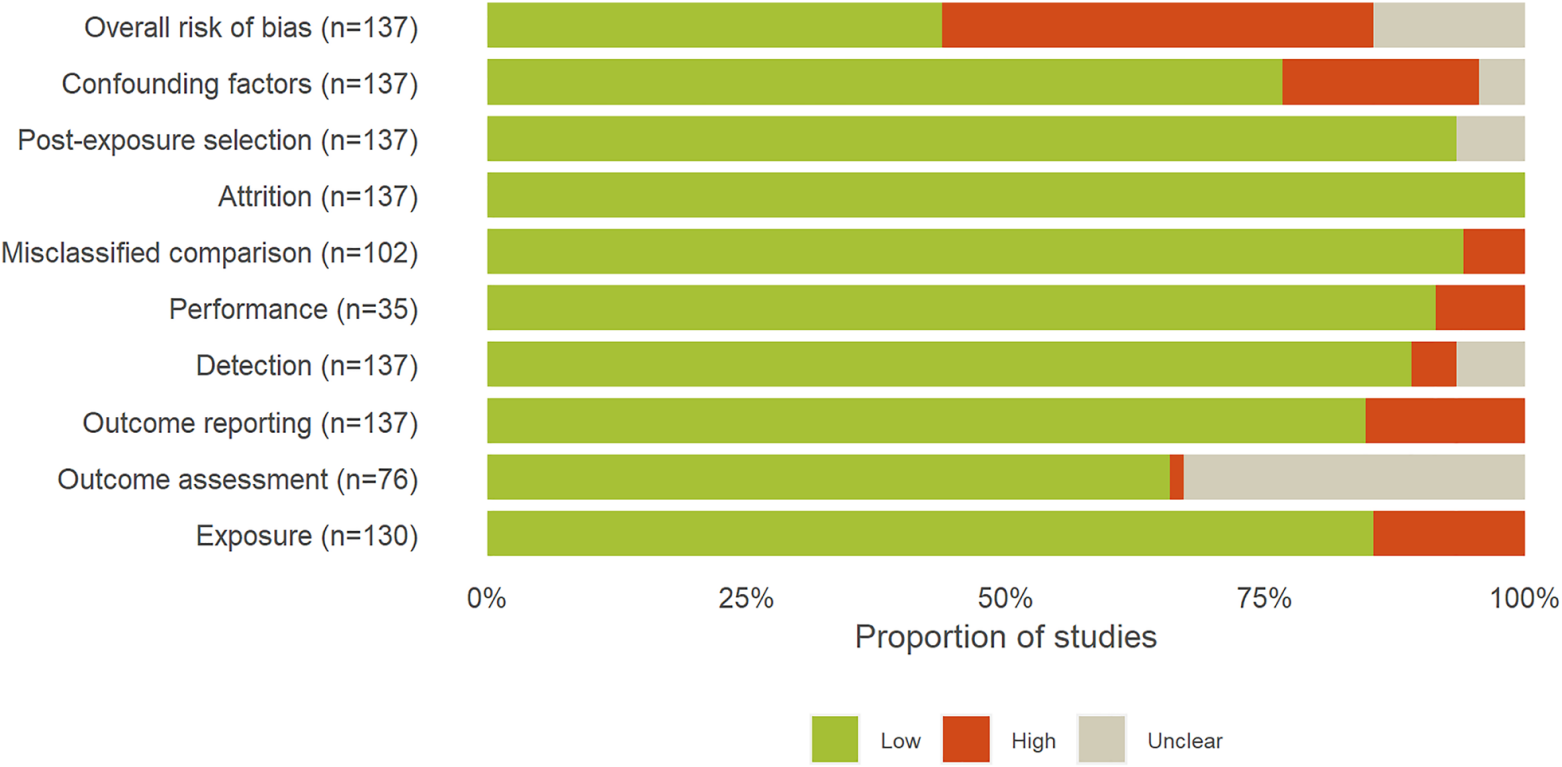
Proportions of included studies by overall risk of bias and detailed results for each criterion. Note that the total number of studies assessed for each criterion differs as: only observational and modelling studies were checked for the misclassified comparison criterion; only experimental studies were checked for the performance criterion; only studies carrying out a statistical analysis were checked for the outcome assessment criterion; modelling studies were not checked for the exposure criterion

After screening, accepted primary research and modelling articles were split into 142 studies corresponding to one experimental design—i.e. one exposure and one comparator. Each study was subjected to critical appraisal, except for five which were found to be redundant—same study but reported in several different articles. On the 137 remaining studies, 60 were rated with a low (43.8%), 57 with a high (41.6%) and 20 with an unclear overall risk of bias (14.6%) (Fig. 6). The majority of studies were observational (95 studies, 69.3%) while 35 were experimental studies (25.6%) and 7 modelling studies (5.1%). Overall, the confounding factors criterion was the one with the most high-rated risks of bias (26 studies, 19.0%) followed by the outcome reporting criterion (21 studies, 15.3%) and the exposure one (19 studies, 14.6%), the latter of which was only assessed for the 130 observational and experimental studies—indeed, for the seven modelling studies, PV panels were neither judged to be real nor simulated but modelled. The outcome assessment criterion was only assessed for the 76 studies which carried out a statistical analysis, and was the one most often rated with an unclear risk of bias (25 studies, 32.9%), notably due to the low reporting of statistical assumptions checking. The detailed results of internal validity assessment are provided in Additional file 3.

#### Geographical distribution of included observations

**Fig. 7:**
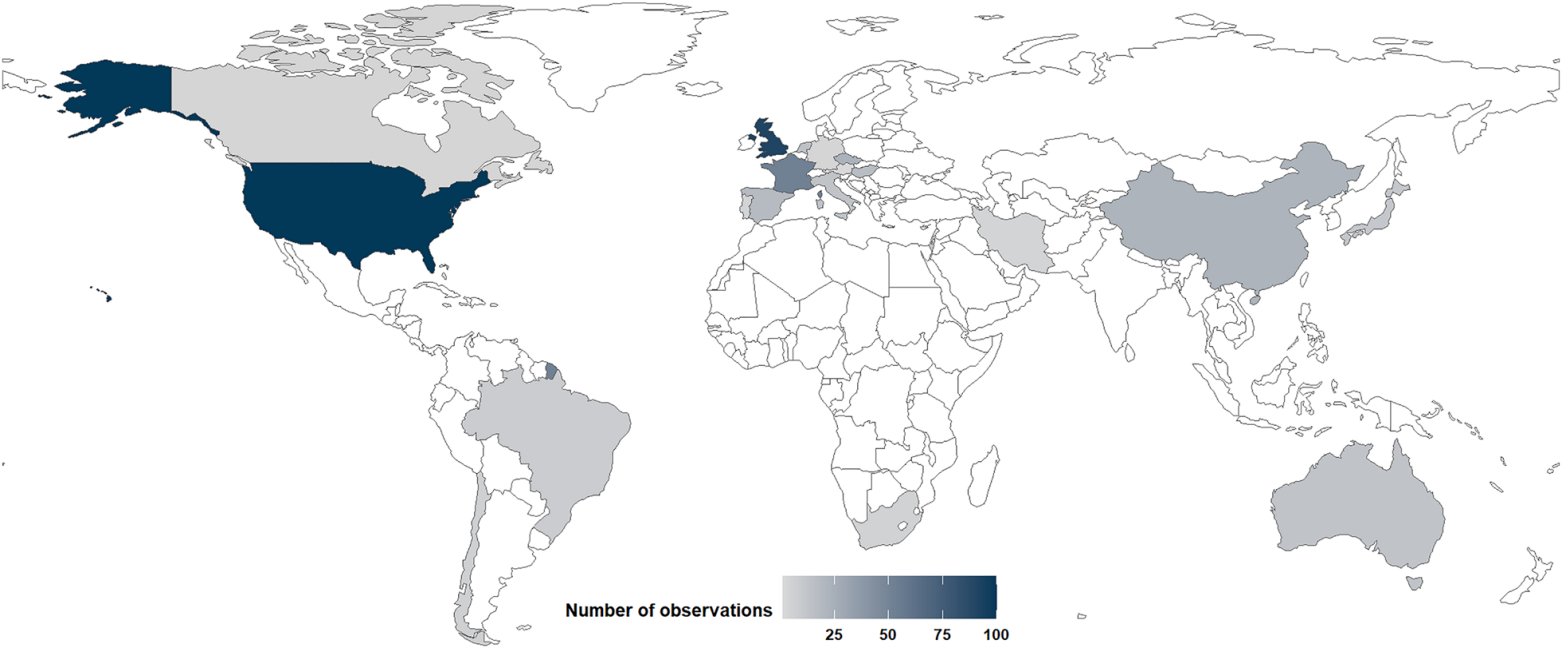
Geographical distribution of observations. For clarity, observations carried out in several locations are not shown: United States | India | Kuwait | United Arab Emirates | Germany (4 observations); United States | India | Kuwait | United Arab Emirates (3 observations); Japan | South Korea (2 observations); Norway | Antarctica (2 observations); United States | Spain (2 observations). See Additional file 5 for more detailed results

After carrying out critical appraisal, the 137 non-redundant studies were further split into 434 observations—i.e. corresponding to one population and one outcome. The United States were the primary research location with 100 observations (23.0%), closely followed by the United Kingdom with 91 observations (21.0%) and then France with 52 observations (12.0%) (Fig. 7 and Additional file 5). When only considering observations from online publication databases and Google scholar, the Czech Republic becomes the third most studied location with 23 observations (6.4% of the 359 observations in this subset) and France, joint ninth with Japan and Israel, with just 11 observations (3.1%). This highlights a bias towards French grey literature as the call for grey literature was primarily answered by French researchers—41 observations out of 63 provided by grey literature were from articles written in French. Overall, observations were mainly carried out in a temperate climate (280 observations, 64.5%), arid climate (68 observations, 15.7%) or in a mix of both climates (30 observations, 6.9%) (see Additional file 5).

### Mapping the quantity of observations on the effects of PV installations on terrestrial biodiversity

Observations were mostly made in-situ (381 observations, 87.8%) with 48 observations carried out on the specific context of green roofs (11.1%). We collected 37 observations (8.5%) which were conducted at a large scale (e.g. at a regional, country or global scale), mainly on outcomes related to land use, habitat alteration, or habitat connectivity. Only 4 observations were made ex-situ (0.9%), all on a miniature USSE facility situated on a building roof.

#### Studied taxa

**Fig. 8:**
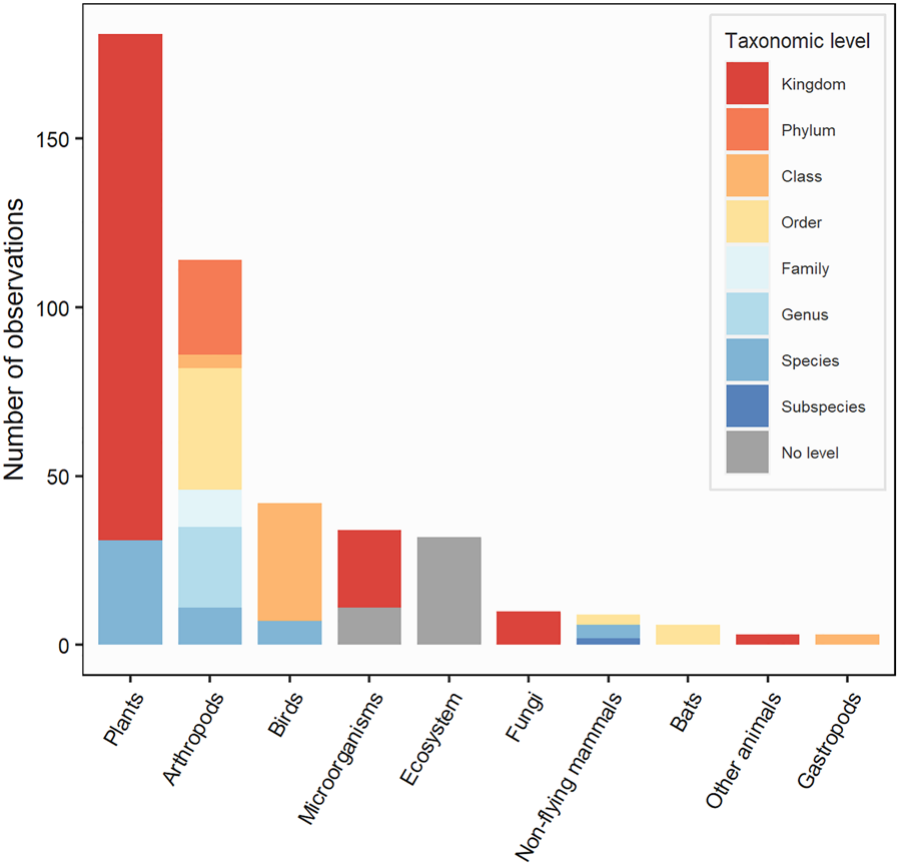
Number of observations by taxa and taxonomic level. The ‘Ecosystem’ population was used for observations carried out at the level of the ecosystem: i.e. on ecosystem fluxes, land use or habitat alteration

Overall, 51 different taxonomic units were recorded but higher levels of taxonomic classification predominated with taxa mainly classified at the kingdom level (186 observations, 42.9%) (Fig. 8). Plants were the most studied taxon with 181 observations (41.7%), followed by arthropods with 114 observations (26.3%) and birds with 42 observations (9.7%). Microorganisms were then studied in 34 observations (7.8%), mainly at the kingdom level. A proportion of observations were also carried out at the ecosystem scale (32 observations, 7.4%).

#### Type of exposure

**Fig. 9:**
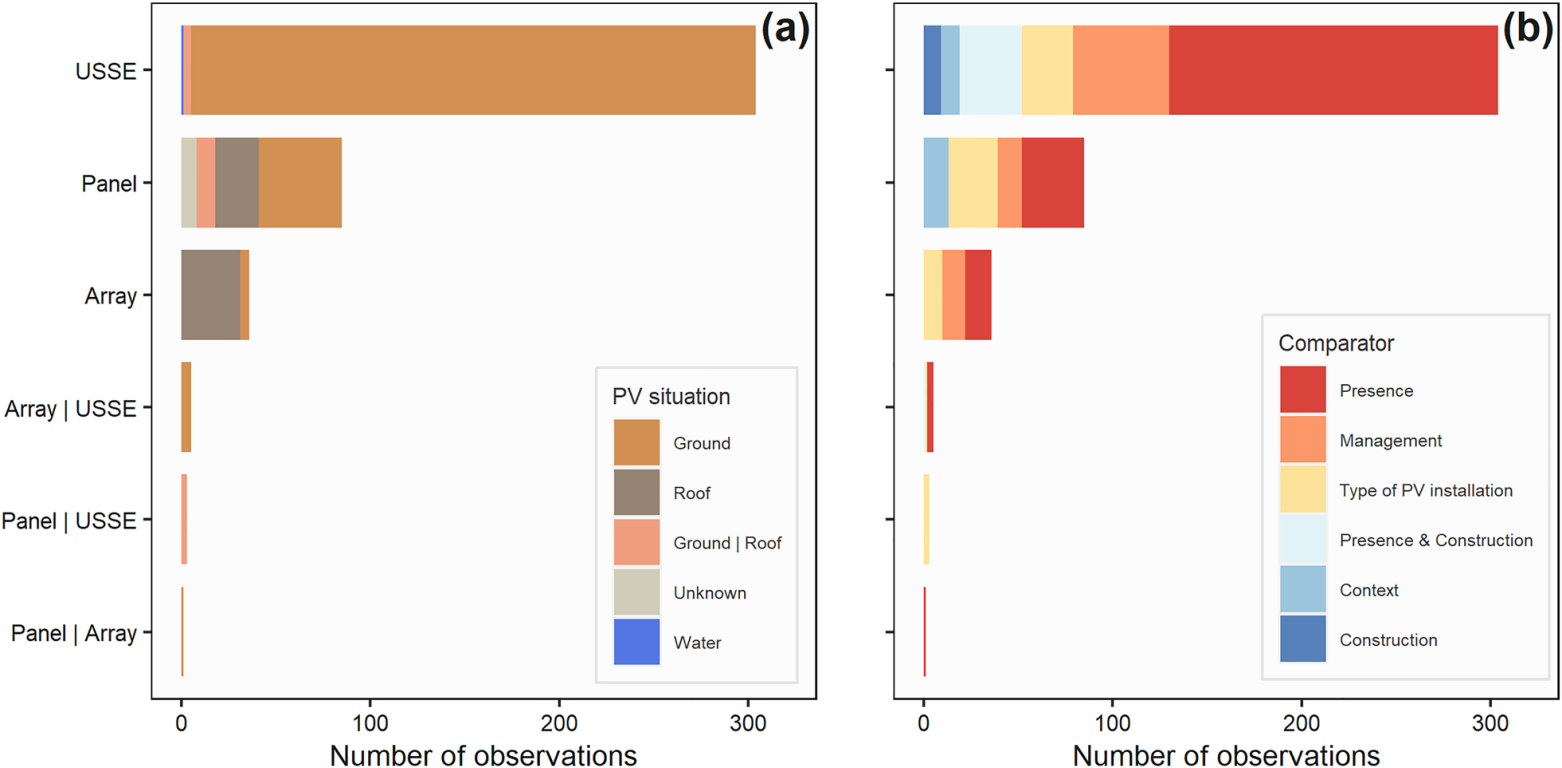
Number of observations by type of PV installations and **a** situation of PV installations or **b** type of comparator. *PV* Photovoltaic and solar thermal; *USSE* Utility-Scale Solar Energy. The pipe | signifies observations carried out on several types of PV installations or several types of PV situations

Observations were overwhelmingly carried out on ground-mounted USSE facilities (304 observations, 70.1%), then on panels either on the ground (44 observations, 10.1%) or on roofs (23 observations, 5.3%) (Fig. 9a). Arrays were mainly studied in the context of roofs (31 observations, 7.1%). Only one observation of floating PV installations was included in this map. We were not able to include any observation on solar thermal panels. The PV panel technology was hardly ever stated (unknown in 81.1% of cases) but 43 observations were carried out, at least in part, with simulated PV panels (9.9%), 29 with mono- or poly-crystalline (6.7%), 9 on thin-film (2.1%) and one with both thin-film and crystalline technologies (Table 3). In the specific case of the 304 observations on USSE facilities, the presence of a sun-tracking system was mostly unknown (46.4%)—which may thus possibly be assumed as not present. If recorded, USSE facilities used more often sun-tracking systems (24.3%) than fixed-tilt arrays (20.7%). USSE facilities were also mostly fenced (56.9%) but, for a substantial proportion of observations, the presence of security fences was unknown (103 observations, 33.9%). Information on the type of management practices implemented at USSE facilities was often provided (53.0%) and included grazing, mowing or herbicide-spraying practices—however, for 130 observations, management practices were left unknown (42.8%).

**Table 3:** Number of observations for several characteristics of PV installations

		Number of observations	Proportion of observations
PV panel technology	Unknown	352	81.1%
	Simulated	43	9.9%
	Crystalline	29	6.7%
	Thin-film	9	2.1%
	Crystalline | Thin-film	1	0.2%
	Thermal	0	0%
USSE facility sun-tracking system	Unknown	141	46.4%
	Sun-tracking	74	24.3%
	Fixed-tilt	63	20.7%
	Both	26	8.6%
USSE facility security fencing	Yes	173	56.9%
	Unknown	103	33.9%
	Both	15	4.9%
	No	13	4.3%
USSE facility management practices	Yes	161	53.0%
	Unknown	130	42.8%
	No	13	4.3%

For the PV technology, ‘Simulated’ includes the values: Simulated and Simulated | Unknown; ‘Crystalline’ includes: Crystalline, Monocrystalline and Monocrystalline | Polycrystalline; ‘Thin-film’ includes: Thin-film, CdTe Thin-film and CdTe Thin-film | Unknown. For sun-tracking system, ‘Sun-tracking’ includes the values: Yes and Yes | Unknown. For security fencing, ‘Yes’ includes the values: Yes and Yes | Unknown

*PV* Photovoltaic and solar thermal; *USSE* Utility-Scale Solar Energy.

#### Type of comparator

Observations mainly investigated the presence of PV installations (225 observations, 51.8%), and more especially USSE facilities (174 observations, 40.1%) (Fig. 9b). In this particular case, controls mainly consisted in open areas within USSE facilities but not between rows of PV panels (115 observations, 26.5%), areas between PV panels (28 observations, 6.5%) and/or reference areas outside USSE facilities (14 observations, 3.2%). The effect of management practices at PV installations was the second most studied comparator with 76 observations (17.5%) followed by comparisons between different types of PV installations with 67 observations (15.4%). The concomitant effects of the construction and presence of PV installations was studied in 34 observations (7.8%) while the sole effect of construction in 9 observations (2.1%), both types of comparators were assessed with BAE-type (41 observations, 9.5%) and BACE-type designs (2 observations, 0.5%). Finally, 23 observations investigated the effect of the context in which PV installations are located (5.3%).

#### Measured outcomes

**Fig. 10:**
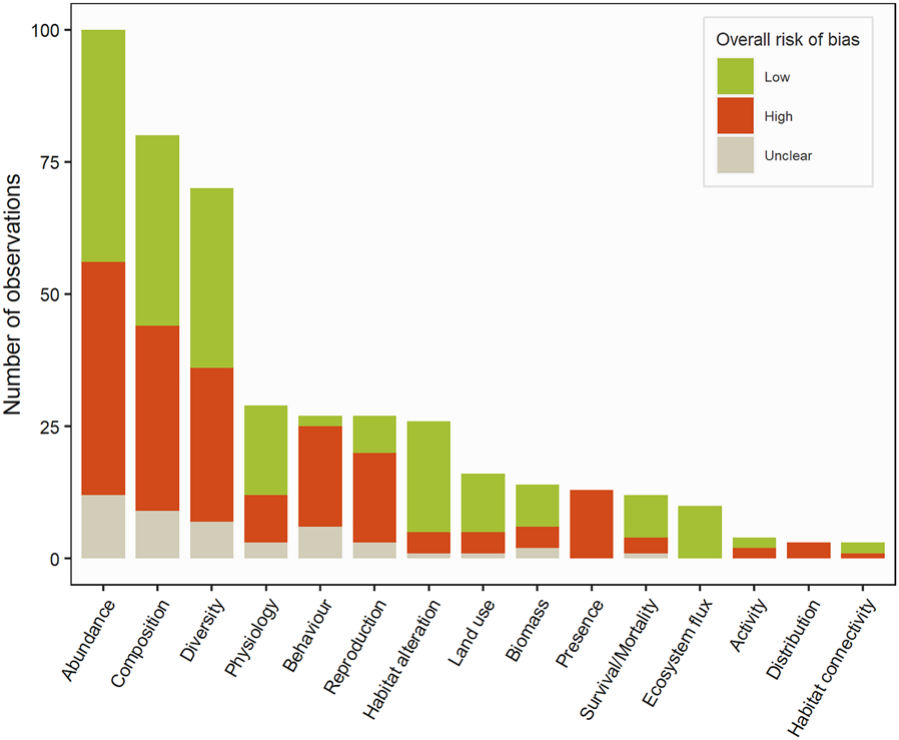
Number of observations by outcome and internal validity assessment result

Overall, 15 different types of outcomes were recorded (Fig. 10). Species abundance, community composition and species diversity were the three most studied outcomes with respectively 100 (23.0%), 80 (18.4%) and 70 observations (16.1%). As species behaviour and reproduction outcomes were more often investigated on simulated PV panels (with respectively 14 and 12 observations, corresponding to 51.9% and 44.4% of observations for each outcome), they both showed a higher proportion of observations rated with an overall high risk of bias when compared to other outcomes. Observations assessing the presence of certain species were exclusively rated with a high risk of bias and consisted of assessments of PV panel soiling by inorganic and organic particulate matter (i.e. presence of bacteria or fungi on PV panels) or opportunistic fauna or flora surveys carried out during the construction of USSE facilities.

### Identified knowledge clusters

#### What is the effect of PV installations on plant communities?

**Fig. 11:**
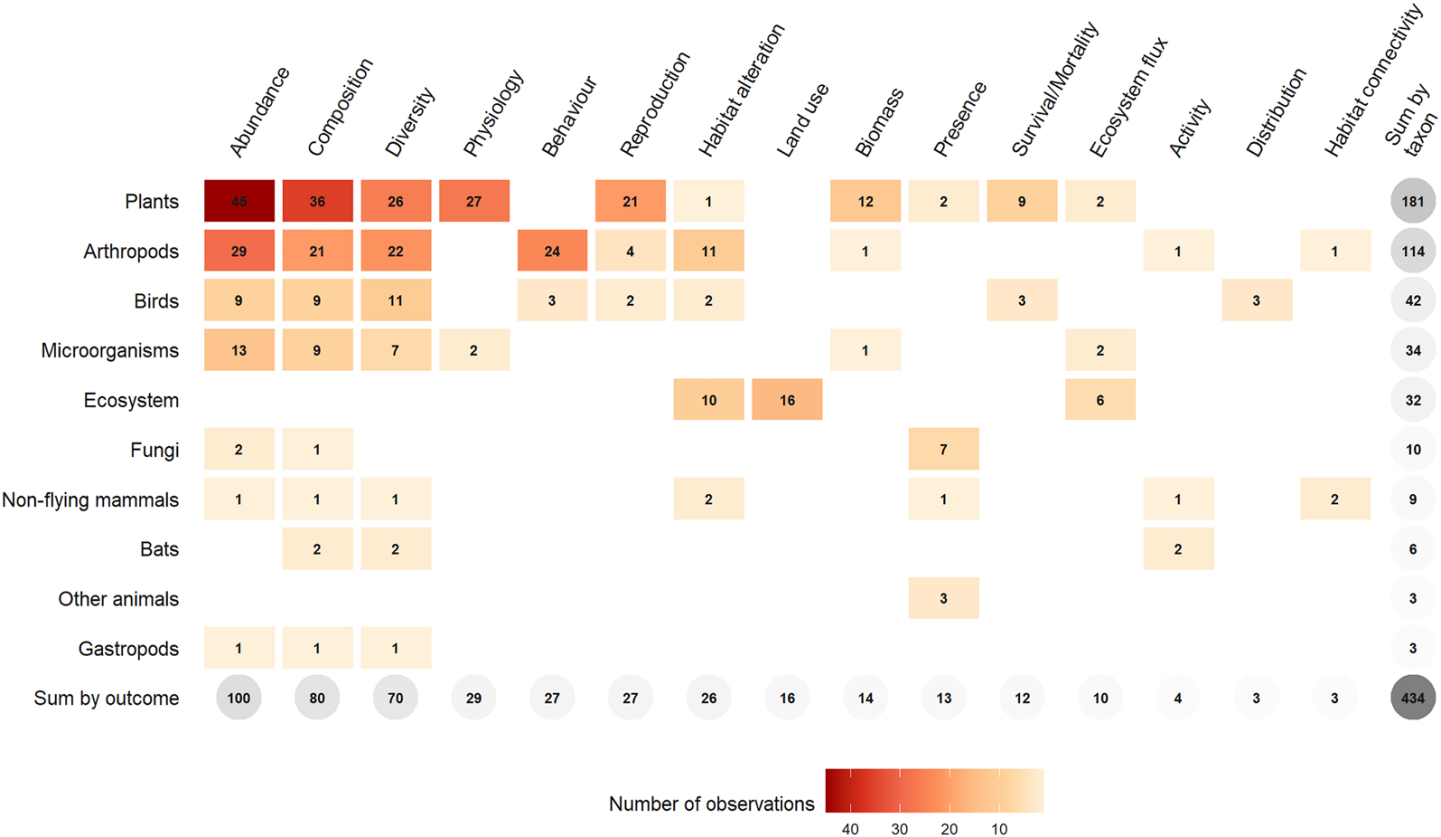
Heat map of observations by taxa and outcome

**Fig. 12:**
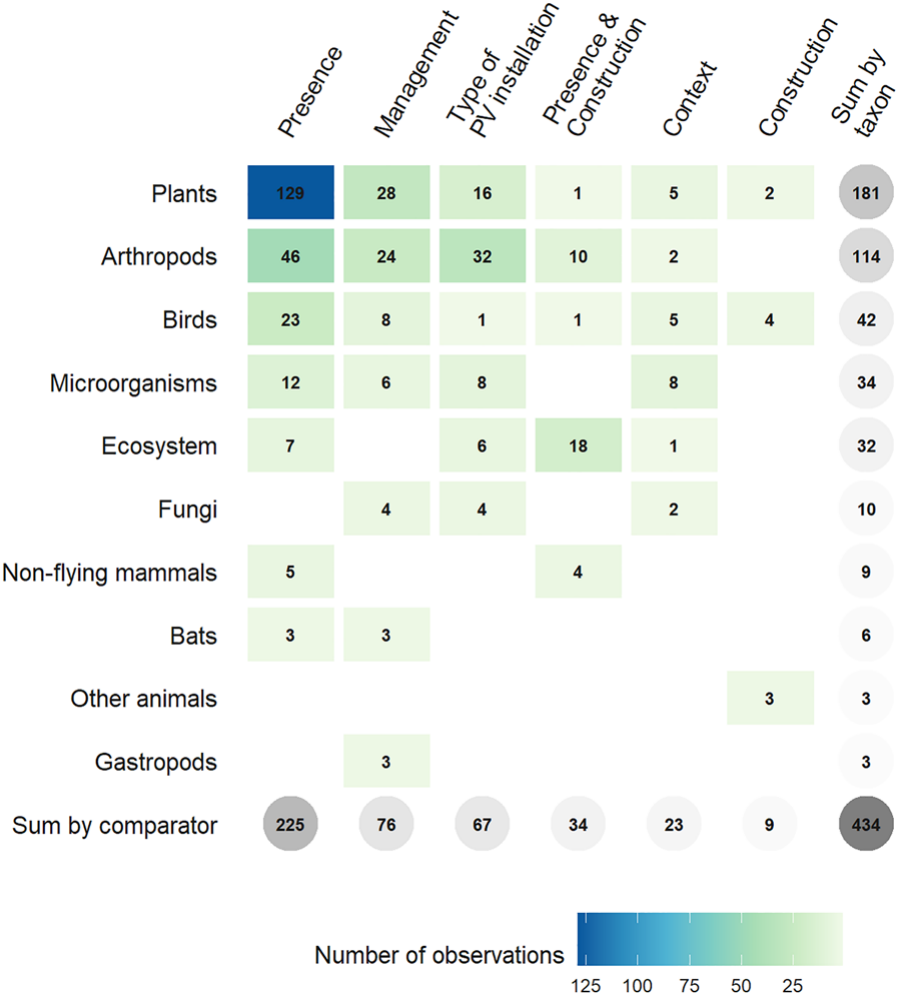
Heat map of observations by taxa and type of comparator

The largest knowledge cluster concerned the effect of PV installations on plant communities with 181 observations (41.7%)—corresponding to 41 articles—and more especially the effect of their presence with 129 observations (29.7%) (Fig. 11, Fig. 12). These observations often consisted in comparison of plant communities living under and between PV panels as well as in open areas within or outside USSE facilities. Then, a number of observations also investigated the effect of management practices and types of PV installations on plants with, respectively 28 and 16 observations (6.5% and 3.7%). Plant abundance was the most commonly studied outcome with 45 observations (10.4%), followed by plant composition with 36 observations (8.3%), plant physiology with 27 observations (6.2%), plant diversity with 26 observations (6.0%), and plant reproduction with 21 observations (4.8%). Physiological outcomes mostly consisted in measures of plant height and growth while reproductive ones mainly studied the seed bank of desert plant species under PV panels. Based on this first cluster, a systematic review could thus focus on disentangling the effects of PV installations, and especially their presence, on plant communities.

#### What is the effect of PV installations on arthropod communities?

Another important knowledge cluster dealt with the effect of PV installations on arthropod communities with 114 observations (26.3%)—corresponding to 25 articles—and with a clear focus on pollinators (Fig. 11)—at least 52 observations (12.0%) when only considering bees, bumblebees, and butterflies. The effect of the presence of PV installations on arthropods was more often investigated with 46 observations (10.6%) and mostly consisted of designs comparing arthropod communities between PV panels and in open areas within or outside USSE facilities (Fig. 12). Next, observations comparing arthropod communities between different types of PV installations (32 observations, 7.4%) and investigating the effect of management practices (24 observations, 5.5%) were the most represented. In the case of arthropods, outcomes were mainly focused on species abundance (29 observations, 6.7%), behaviour (24 observations, 5.5%), species diversity (22 observations, 5.1%) and community composition (21 observations, 4.8%). Measures of arthropod behaviour mainly focused on the attraction of aquatic insects and tabanids towards varying types of PV panels (e.g. coatings, gridding patterns, underlying surfaces). Although the literature is slightly more heterogeneous for arthropods, a systematic review investigating the effects of PV installations on arthropod communities could also be contemplated, maybe particularly focusing on the presence of PV installations and specific outcomes such as species abundance and diversity as well as community composition.

#### At a larger ecosystem scale, what is the effect of PV installations on overall species abundance?

**Fig. 13:**
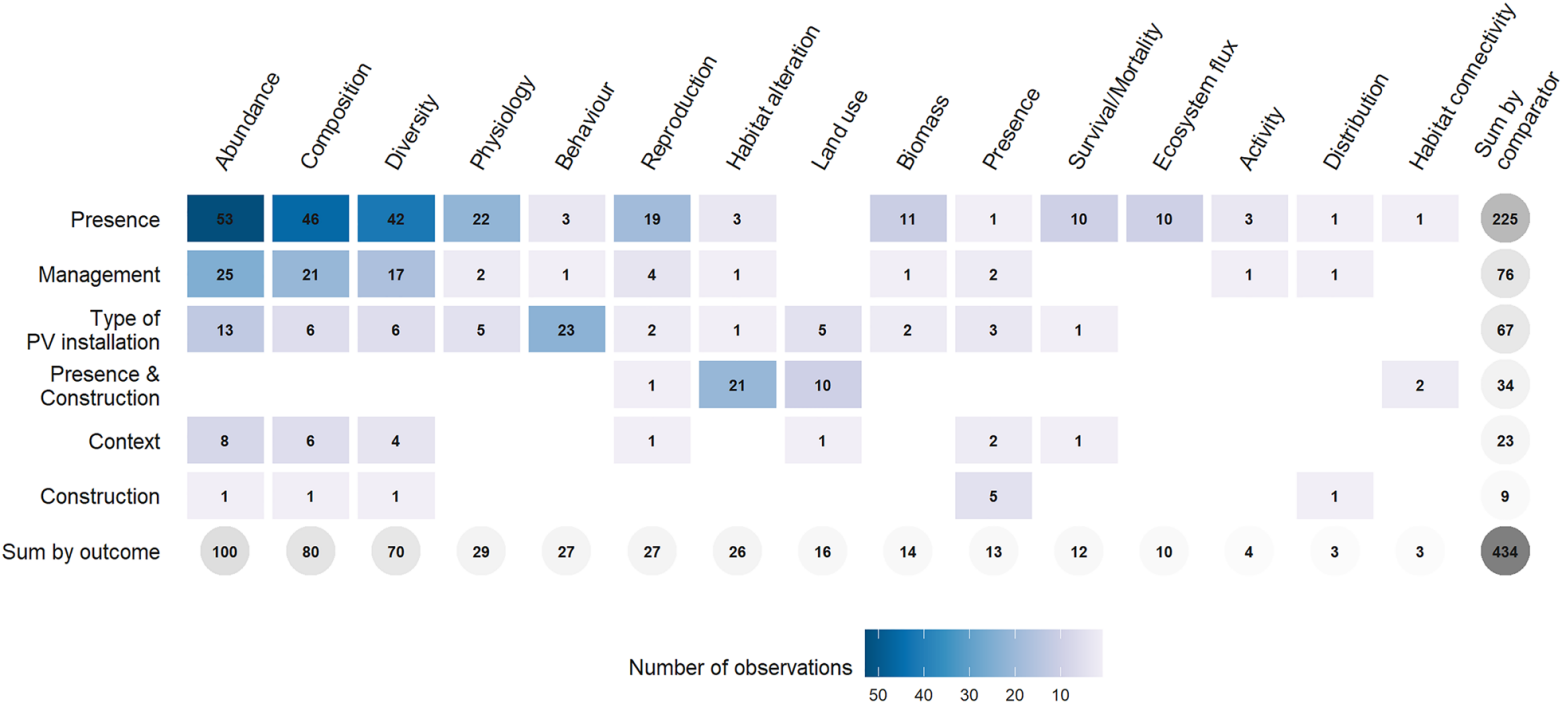
Heat map of observations by type of comparator and outcome

A last cluster of observations should be highlighted and concerned the overall effect of PV installations on species abundance with 100 observations (23.1%) (Figs. 11, 13)—corresponding to 46 articles. Observations mostly investigated the effect of the presence of PV installations (53 observations, 12.2%) and then of management practices (25 observations, 5.8%). This last knowledge cluster could be the focus of another systematic review which would be aimed at estimating the overall effect of PV installations on species abundance. While not crossing the 100-observation threshold, community composition and species diversity outcomes could also be featured in this systematic review as they were both studied quite substantially with 80 (18.4%) and 70 observations (16.1%) respectively.

### Identified knowledge gaps

#### Geographical regions

The majority of observations were made in the most developed countries (Fig. 7) but some regions with high solar potential and resources such as Africa, Southeast Asia or South America [68] have been much less studied thus far (see section ‘Limitations of the map’ for further explanations regarding the potential geographical biases of this map).

#### Populations

While a wide range of terrestrial species have already been investigated, additional research on birds could be undertaken in order to improve our knowledge on their potentially deadly interactions with PV installations [22, 33, 46]. As non-flying mammals were found to be very little studied (9 observations, 2.1%), they, however, could be potentially heavily impacted by new developments of PV installations. For instance, PV installations have already been shown to affect habitat connectivity for some species of large mammals such as the Florida panther *Puma concolor* and the pronghorn *Antilocapra americana* [21, 22]. Amphibians or reptiles have never been investigated to date which could represent a major knowledge gap, especially so when PV installations are increasingly being built in deserts and wetlands, two natural habitats potentially inhabited by both of these taxa [38]. While PV installations may influence bat foraging behaviour—as PV panels may attract some insect species which can represent potential prey for bats [27, 32]—or induce habitat loss due to avoidance by bats [69, 70], very few studies on chiropterans were recorded so far (6 observations, 1.4%). Most observations on microorganisms were carried out on bacteria and fungi found on PV panels but fewer observations investigated the effects of PV installations on soil microorganisms and fauna. However, to become a functional semi-natural ecosystem capable of hosting a wide array of interacting plant and animal species, PV installations should first represent a suitable habitat for an abundant and diverse soil biological community to thrive (e.g. bacteria, fungi and fauna) [31]. As a whole, more observations investigating the effects of PV installations on ecological functions should be carried out in order to better take into account the effects of PV installations on more common or unprotected wildlife.

#### Exposures

Only one observation on floatovoltaics was included in this map and dealt with the land sparing opportunity of such installations [71]. Their impacts on terrestrial (e.g. birds, bats) or semi-aquatic (e.g. aquatic insects, amphibians) species have thus yet to be studied. However, apart from potential direct impacts, the hypothesised changes in primary production caused by the reduction of photosynthetically active radiations and temperatures under floating PV installations [9, 72, 73] may also indirectly impact these species. It should also be noted that, as aquatic species were excluded from this map, no conclusions can be drawn on the state of the available knowledge regarding the impacts of floatovoltaics on these organisms, even though some studies could already be available on the matter.

Based on our initial results, the effects of solar thermal panels on wildlife and ecosystems have yet to be studied (but see section ‘Limitations of the systematic map method’ for potential limitations of our search string). However, it remains to be elucidated whether their potential impacts could be similar to the ones observed in the case of PV panels.

#### Comparators

The effect of the presence of PV installations (225 observations, 51.8%) was most often studied while fewer observations investigated the varying management practices implemented at USSE facilities (76 observations, 17.5%)—e.g. mowing, grazing, rehabilitation. Likewise, comparisons between different types of PV installations (e.g. scale, inter-row width, height, angle, tracking system, technology) were more rarely conducted (67 observations, 15.4%). Comparisons between PV installations located in different contexts (e.g. previous land use, surrounding habitats, climates) were also only assessed in 23 observations (5.3%). Finally, Presence & Construction or Construction comparators were investigated in 43 observations (10.0%) but many more studies built on BDAE-type experimental designs should be carried out to more accurately estimate the impacts of PV installation construction or even dismantlement on species and ecosystems.

#### Outcomes

While species abundance, community composition and species diversity were the most commonly assessed outcomes, the direct effects of PV installations on animal mortality (12 observations, 2.8%) or, at a larger scale, on species distribution or habitat connectivity were not often studied (both 0.7% with 3 observations). While arthropod behaviour has already been studied quite substantially, on a higher level of the food web, bird (0.7%, 3 observations) and bat behaviour (not studied) have been too scarcely assessed thus far. Finally, outcomes related to animal reproduction (6 observations, 1.4%) or physiology (not studied) were also poorly studied. However, bird may, for instance, nest underneath PV panels and could then be subjected to potential adverse effects due to PV panels themselves or because of the management practices implemented at USSE facilities [74].

### Limitations of the map

#### Limitations of the systematic map method

The first limitation of this systematic map concerns the search for literature. The term ‘renewable energy’ was not used in the search string as it was deemed too broad for the scope and duration of the mapping process. While this term could have made our search too noisy, some relevant citations may still have been missed. Indeed, some citations identified and added by the review team only used the key term ‘renewable energy’ in their titles, abstracts or keywords (topic ‘TS’) and were therefore not retrieved by our search on online publication databases. However, as we extensively tested our search string prior to retrieving any citation, we believe that our corpus should be comprehensive and missed citations limited. Likewise, the search term ‘solar thermal’ was also excluded while building our search string because it resulted in too many citations referring to Concentrated Solar Power, an exposure excluded from this map. Nevertheless, citations referring to thermal solar panels were already retrieved by our actual search string while the plurality of literature sources being investigated may also have enabled most of the citations relative to solar thermal panels to be found. As an important number of reviews and meta-analyses were retrieved, a relevant step would have been to perform forward and backward citation chasing—exploration of citations from the literature collated in our final systematic map corpus [75]—but it was not possible in this systematic map because of time constraints.

While we had to exclude articles not written in English and/or French based on the linguistic competences of the review team, this could have precluded us from retrieving more references from locations where publications may not usually be published in English (e.g. China, Japan, Russia, India or Spanish-speaking countries). As such, 42 potentially relevant citations were excluded at full-text screening (see Additional file 2). In addition, the search for grey literature provided a substantial number of observations but mainly in the context of France—41 observations out of the 63 provided by grey literature were from articles written in French. A more thorough and international call for grey literature may have yielded many more references from all around the world, and notably from countries where PV installations are currently being built, but where the published research may not yet have caught up (see below section ‘Limitations of the evidence base’).

A substantial amount of citations screened on title and full-text (approximately 90%) were only assessed by one reviewer while the CEE advocates for a full double-screening of citations and we therefore cannot completely rule out that screening errors occurred—estimated at 8% in CEE guidelines [47]. To avoid any significant bias, we checked screener agreement by computing Randolph’s κ coefficient and obtained excellent agreement rates at each screening steps (overall, approximately 0.8). Similarly, the majority of metadata extraction and critical appraisal have been carried out by one reviewer (approximately 80%). However, we ensured that a maximum level of agreement between reviewers—and therefore the lowest level of errors—was reached by carrying out agreement tests at the beginning and end of both steps.

#### Limitations of the evidence base

While distribution of most studied countries and total installed capacity of PV by country seem to be generally in line (Fig. 7, Table 4), some countries were heavily under-represented relative to their PV capacity, namely Germany, India, Viet Nam or Poland. On the contrary, some other countries, where comparatively less PV have been installed so far, have been the subject of a far more intense research effort, namely Czech Republic, Hungary, Israel, Chile or South Africa. In particular, the United Kingdom is the second most studied location but the fourteenth country by installed PV capacity while China has, by far, the largest installed PV capacity in the world but is only the fifth location by number of observations. These geographical biases highlighted in this map may either be explained: by the relatively lower presence of PV installations in less studied regions, by a lesser research effort on ecological matters in some countries, by the fact that articles published in some languages have been excluded from this map, or alternatively because grey literature from certain countries can be more difficult to access—notably, in the context of this map, because of the full-text screening language criterion and because our call for grey literature was mainly answered by French researchers. Consequently, as most observations were carried out in a temperate climate, generalising the reported effects of PV installations on the whole of biodiversity could prove difficult. For instance, plant response to USSE facilities may very well vary according to the climatic context of observation—plant biomass may be lower under PV panels in temperate climates but greater in arid ones for example [23, 37].

**Table 4:** Number of observations and installed capacity of PV by country

	Number of observations		Installed PV capacity (MW)
USA	100	China	392,436
UK	91	USA	111,535
France	52	Japan	78,833
Czech Republic^#^	23	Germany^*^	66,552
China	22	India^*^	62,804
Spain	18	Australia	26,789
Hungary^#^	17	Italy	25,077
Netherlands	14	Brazil	24,079
Australia	13	Netherlands	22,590
Italy	13	South Korea	20,975
Israel^#^	11	Viet Nam^*^	18,474
Japan	11	Spain	18,214
Brazil	8	France	17,410
Chile^#^	6	UK	14,412
South Africa^#^	5	Poland^*^	11,167

*PV* Photovoltaic; *USA  *United States of America; *UK *United Kingdom

# highlights countries which have been the subject of a more intense research endeavour comparatively to their lower installed PV capacity

* highlights countries which have been the subject of a less intense research endeavour (no observations found in this map) comparatively to their higher installed PV capacity

Only the top 15 most studied countries found in this map are presented; similarly, only the top 15 countries with the largest installed PV capacity are presented. Installed PV capacity are derived from the IRENA (International Renewable Energy Agency) [4]

Concerning the designs of PV installations, very little data was usually provided by authors on: the technology of PV panels, the presence of sun-tracking devices and security fences or the type of management practices implemented at USSE facilities. Almost 10% of observations were carried out on simulated PV panels, often investigating plant reproduction under wooden panels covered by plastic sheeting [58, 76, 77] or aquatic insect and tabanid attraction towards highly polarizing surfaces such as black plastic trays [26, 32, 78]. Based on the exposure criterion of our critical appraisal tool, these experiments were rated with a high risk of bias—14.6% of the 130 observational and experimental studies. Indeed, we considered they were more prone to bias due to potential high levels of confounding variables, particularly with respect to differences of microclimatic conditions and reflection-polarisation characteristics between simulated and real PV panels. Concerning the other results of internal validity assessment, a substantial number of studies was subjected to bias due to confounding factors (19.0%) and outcome reporting (15.3%). It also appeared that a third of studies carrying out a statistical analysis (32.9%) did not specify their assumptions and were rated with an unclear risk of bias. Therefore, to facilitate and improve the quality of this crucial step of systematic evidence syntheses that is critical appraisal, we advise for an overall better reporting of methods and statistical analyses.

Finally, more robust experimental protocols such as Before-After-Control-Exposure (BACE) experimental designs were only conducted twice (0.5% of all observations)—one to assess changes in vegetation cover before, during, and after the construction of a USSE facility as well as in a reference area by using satellite imagery [79]; the other to observe the dynamics of golden eagle *Aquila chrysaetos* populations located near or far from a USSE facility during and after its construction [80]. Furthermore, some observations which assessed the effects of the presence of PV installations on plant communities only compared plots located between and under PV panels. However, areas between PV panels may not represent fully adequate controls as they may be half-shaded by PV panels—depending on PV panel inter-row width and on the changing orientation of the sun throughout the day—or because vegetation management practices may not be strictly similar in between areas compared to areas directly below PV panels (e.g. grazing, mowing), or also because of lasting consequences of previous construction works. As such, experimental plots investigating plant communities located in open areas within USSE facilities could serve as stronger controls since they should be under no influence from PV panels whatsoever. In addition, to accurately estimate the effects of management practices or previous construction works, studies built on more robust BACE experimental designs could notably be carried out, or alternatively/complementarily reference plots located outside USSE facilities, in a ‘pristine’ reference area—i.e. a wisely-chosen habitat comparable to the one which previously existed at USSE facility—could be more often laid out.

## Conclusions

This systematic map aimed at collating the available evidence regarding the effects of PV installations on wild terrestrial and semi-aquatic species. Our search for literature identified 158 relevant articles, including 97 primary research and modelling articles. The latter were split into 137 studies (by experimental designs, i.e. one exposure and one comparator) and further down into 434 observations (i.e. corresponding to one population and one outcome). Three primary knowledge clusters were identified and concerned: (i) the effects of PV installations on plant communities, (ii) their effects on arthropod communities and finally (iii) their effects, at a larger ecosystem scale, on overall species abundance.

### Implications for research

First, concerning this systematic map, it has to be noted that the literature search was mainly performed in June 2022 but, based on the current context of a thriving solar energy industry and the resulting urgent need for a better understanding regarding the effects of PV installations on biodiversity, we expect many more publications to be available in the coming years. We therefore advise for this systematic map to be updated in a two-to-three-year timeframe. For now, this systematic map has already led to the identification of three knowledge clusters regarding the effects of PV installations on plant and arthropod communities as well as their effects at a larger ecosystem scale on overall species abundance. These areas of research seem already sufficiently well studied for potential systematic reviews to be contemplated and, as internal validity has already been assessed in this map, a substantial amount of time will hopefully be saved when synthesising the studies that will be included in these future systematic reviews.

Concerning the actual state of the literature regarding the effects of PV installations on biodiversity, while a wide range of different taxa has already been investigated, very little research has thus far been conducted on non-flying mammals and bats. However, USSE facilities have already been shown to influence the spatial distribution and habitat connectivity of large mammals [21, 81] and induce habitat loss due to potential avoidance by bats [69, 70]. Photovoltaic panels might also represent sensory traps for bats and lead to potential collisions as it has already been found in the case of smooth black vertical surfaces [82]. No study has been produced thus far on reptiles and amphibians but these two highly-sensitive taxa should pressingly be the subject of new investigations. In addition, most observations studied microorganism populations found on PV panels but the effect of PV installations on soil microorganisms and fauna was far less investigated. More studies elucidating the effects of PV installations on ecological functions should also be conducted. Finally, the potential habituation of species to PV installations could also represent a novel research topic worthy of further investigations. Likewise, the potential of USSE facilities to promote the spread of invasive alien species should be further clarified, notably as the construction of these installations may create newly disturbed habitats conducive to the proliferation of such species.

While PV installations and especially ground-mounted USSE facilities have been the subject of most research, the impacts of solar thermal panels on wildlife and ecosystems have yet to be studied. Thus, it remains to be found whether these impacts could be similar to the ones observed in the case of PV panels. Moreover, it appeared that the effects of floating PV installations on terrestrial or semi-aquatic species have never been studied yet. However, floatovoltaics may have similar direct impacts on species as ground-mounted USSE facilities. In addition, floating PV installations may also change the overall functioning of aquatic habitats by reducing photosynthetically active radiations and temperatures, thus altering the primary production of the ecosystem as a whole [9, 72, 73], which underlines the urgent need for further research to be carried out on the matter.

Moreover, more comparisons between different types of PV installation designs (e.g. scale, inter-row width, height, angle, tracking system, technology) should be carried out. In particular, it remains to be elucidated which alternative between ‘land sparing’ or ‘land sharing’ should be preferred in order to reduce the effects of PV installations on biodiversity—should USSE facility land-use be kept to a minimum to avoid fragmenting ecosystems (high packing factor and small inter-rows) or should USSE facilities be treated as potentially relevant habitats for hosting wildlife and be therefore integrated within local ecological networks (which presupposes lower packing factor and wider inter-rows). As such, new research could also try to determine the potential cumulative effects of building several PV installations at the landscape scale—or even mixed with several other types of renewable energy installations such as wind turbines.

Experimenting on the design of PV installation may often prove difficult as, for instance, USSE facilities with varying specificities can be located far away, which can therefore introduce confounding factors potentially altering the validity of final results—e.g. surrounding habitats, climatic conditions. As such, the creation of an experimental USSE facility with different specificities within a unique site could potentially present the advantage of reducing the influence of these confounding variables. Overall, researchers should endeavour to build more robust experimental designs such as complete Before-After-Control-Exposure (BACE) designs in order for more definitive conclusions about the effects of PV installations on biodiversity to be drawn. Therefore, to build these complete and robust experimental designs, researchers need to be provided with exhaustive and transparent information concerning the technical characteristics of the USSE facilities in which they wish to carry out their studies. Researchers should also endeavour to improve reporting, especially in the case of statistical assumptions checking.

Finally, determining which management practices are the most favourable for biodiversity should also be a primary research area. Unfortunately, the effects of the varying management practices being implemented at USSE facilities (e.g. mowing, grazing, rehabilitation, timing) seem to be too rarely studied as of yet. In addition, further experiments should be carried out in as many contexts as possible (e.g. previous land use, surrounding habitats, climates) in order to help better estimate the general effect of PV installations on biodiversity or, on the contrary, to be able to regionalise these effects by biome or grand types of natural habitats.

### Implications for policy/management

The first published article investigating the effects of PV installations on terrestrial biodiversity dated back to 2005 and was a review. However, the first primary research article on the subject was only published in 2010. This five-year delay indicates that, while already needed, no empirical evidence was initially available on this particular subject. Indeed, the first reviews published concerning the effects of PV installations on biodiversity often inferred and extrapolated their impacts from other similar man-made infrastructures built in similar environments [10, 38, 39]. Given that a significant proportion of currently published articles are still reviews or meta-analyses (38.6%), it highlights the remaining knowledge gaps and the significant need for more primary research to be conducted on this research topic.

This systematic map may then represent a potentially relevant first step towards the recognition by practitioners and decision-makers of the need for more research to be undertaken regarding the effects of PV installations on biodiversity. In particular, they may thus allow researchers to more easily access PV installations, which could then, in turn, help expand the volume of research being carried out on the matter. Indeed, as USSE facilities are often surrounded by security fences, they are usually less easily accessible than other types of renewable energy infrastructures such as wind turbines, which may have contributed to the currently existing gaps in the available evidence. In addition, this systematic map could also be used by practitioners to identify which are the management practices actually being implemented at USSE facilities. By then using these practices in their own installations, they could contribute to the global research endeavour and help further assess the efficiency of new management practices aimed at better protecting wildlife and ecosystems. Lastly, another use of this map could also be to strengthen cooperation between researchers, managers and decision-makers in order to conduct new long-term studies, which may be better suited at disentangling the effects of PV installations on, for instance, ecological successions or wider ecosystem changes.

On the one hand, the solar energy industry is currently thriving with an installed PV capacity increasing almost eight-fold between 2013 and 2022 [4]. On the other hand, based on the results presented in this systematic map, the currently available evidence regarding the impacts of PV installations on biodiversity is still scarce. Thus, further research and syntheses should urgently be produced to provide more accurate and reliable information for managers and decision-makers, which therefore should ensure that future PV installations are developed while limiting as much as possible their impacts on wildlife and natural ecosystems.

## Supplementary Information

**Additional file 1. **ROSES form for systematic map protocol.

**Additional file 2. **Search results and citation title and full-text screening.

**Additional file 3. **Study critical appraisal.

**Additional file 4. **Systematic map database.

**Additional file 5. **Additional bibliometric results.

## Data Availability

The datasets supporting the conclusions of this article are included within the article and its additional files.
