# Supplementary material for: Existing evidence on the effects of photovoltaic panels on biodiversity: a systematic map with critical appraisal of study validity
Source: Environ Evid. 2023 Nov 18;12:25. doi: 10.1186/s13750-023-00318-x (PMC11378773; doi:10.1186/s13750-023-00318-x)
Supplement: Supplementary file 5 — Additional file 5. Additional bibliometric results. [file 13750_2023_318_MOESM5_ESM.docx]

**Existing evidence on the effects of photovoltaic panels on biodiversity: a systematic map with critical appraisal of study validity**

Alix Lafitte^1*^, Romain Sordello^1,a^, Dakis-Yaoba Ouédraogo^1,b^, Chloé Thierry^1,c^, Geoffroy Marx^2,d^, Jérémy Froidevaux^3,4,5,e^, Bertrand Schatz^6,f^, Christian Kerbiriou^3,g^, Philippe Gourdain^1,h^, Christian Kerbiriou^3,h^ & Yorick Reyjol^1,i^

^1^ PatriNat (OFB (Office Français de la Biodiversité) – MNHN (Muséum National d’Histoire Naturelle)), 75005 Paris, France

^2^ LPO (Ligue pour la Protection des Oiseaux), 17300 Rochefort, France

^3^ CESCO (Centre d'Ecologie et des Sciences de la Conservation) UMR 7204, Muséum National d’Histoire Naturelle (MNHN), 75005 Paris, France

^4^ CNRS (Centre National de la Recherche Scientifique), Sorbonne Université, Station Marine, 29900 Concarneau, France

^5^ University of Stirling, Biological and Environmental Sciences, Stirling FK9 4LA, Scotland, United Kingdom

^6^ CEFE, Université de Montpellier, CNRS, EPHE, IRD, 34090 Montpellier, France

* Corresponding author: alix.lafitte@mnhn.fr

^a^romain.sordello@mnhn.fr

^b^dakis-yaoba.ouedraogo@mnhn.fr

^c^chloe.thierry@mnhn.fr

^d^geoffroy.marx@lpo.fr

^e^jeremy.froidevaux@stir.ac.uk

^f^bertrand.schatz@cefe.cnrs.fr

^g^christian.kerbiriou@mnhn.fr

^h^philippe.gourdain@mnhn.fr

^i^yorick.reyjol@mnhn.fr

**Additional file 5. Additional bibliometric results**


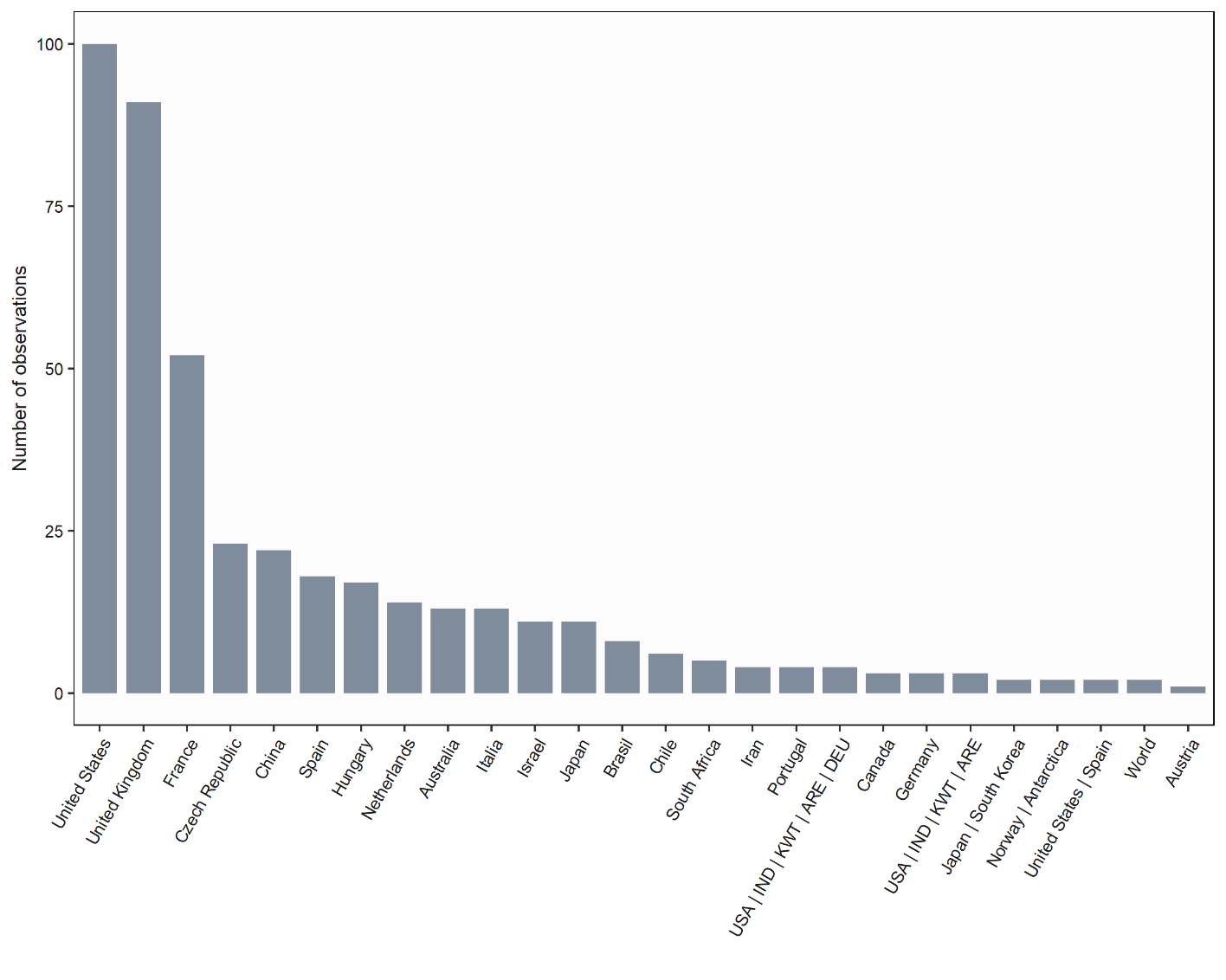


**Figure 1. Distribution of observations by locations.** The pipe | signifies observations were carried out at several locations. ‘USA | IND | KWT | ARE | DEU’ United States | India | Kuwait | United Arab Emirates | Germany, ‘USA | IND | KWT | ARE’ United States | India | Kuwait | United Arab Emirates.


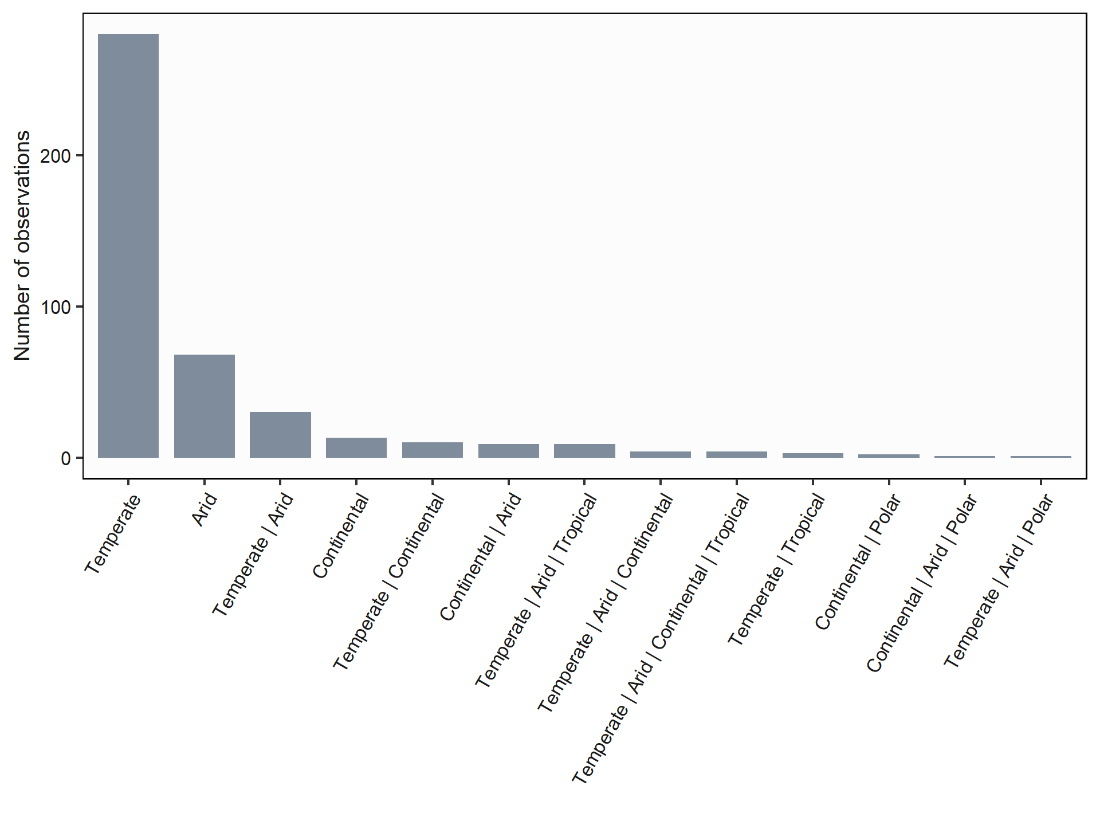


**Figure 2. Distribution of observations by type of climate.** Climatic zones were identified thanks to the Köppen-Geiger climate classification which was displayed on a Google Earth layer^[[1]](#footnote-1)^. The pipe | signifies observations carried out in several types of climates.

1. Rubel F, Brugger K, Haslinger K, Auer I. The climate of the European Alps: Shift of very high resolution Köppen-Geiger climate zones 1800–2100. Meteorologische Zeitschrift. 2017;26:115–25. [↑](#footnote-ref-1)
